# Gene coexpression networks reveal a broad role for lncRNAs in inflammatory bowel disease

**DOI:** 10.1172/jci.insight.168988

**Published:** 2024-02-08

**Authors:** John L. Johnson, Davit Sargsyan, Eric M. Neiman, Amy Hart, Aleksandar Stojmirovic, Roman Kosoy, Haritz Irizar, Mayte Suárez-Fariñas, Won-Min Song, Carmen Argmann, Stefan Avey, Liraz Shmuel-Galia, Tim Vierbuchen, Gerold Bongers, Yu Sun, Leonard Edelstein, Jacqueline Perrigoue, Jennifer E. Towne, Aisling O’Hara Hall, Katherine A. Fitzgerald, Kasper Hoebe

**Affiliations:** 1 Johnson & Johnson Innovative Medicine, Spring House, Pennsylvania, USA.; 2 Department of Genetics and Genomic Sciences, Icahn School of Medicine at Mount Sinai, Icahn Institute for Data Science and Genomic Technology, New York, New York, USA.; 3 Center for Biostatistics, Department of Population Health Science and Policy, Icahn School of Medicine at Mount Sinai, New York, New York, USA.; 4 Program in Innate Immunity, Department of Medicine, University of Massachusetts Chan Medical School, Worcester, Massachusetts, USA.; 5 Immunology Translational Early Development, Bristol Myers Squibb, Summit, New Jersey, USA.

**Keywords:** Gastroenterology, Immunology, Autoimmune diseases, Cellular immune response, Molecular genetics

## Abstract

The role of long noncoding RNAs (lncRNAs) in disease is incompletely understood, but their regulation of inflammation is increasingly appreciated. We addressed the extent of lncRNA involvement in inflammatory bowel disease (IBD) using biopsy-derived RNA-sequencing data from a large cohort of deeply phenotyped patients with IBD. Weighted gene correlation network analysis revealed gene modules of lncRNAs coexpressed with protein-coding genes enriched for biological pathways, correlated with epithelial and immune cell signatures, or correlated with distal colon expression. Correlation of modules with clinical features uncovered a module correlated with disease severity, with an enriched interferon response signature containing the hub lncRNA *IRF1-AS1*. Connecting genes to IBD-associated single nucleotide polymorphisms (SNPs) revealed an enrichment of SNP-adjacent lncRNAs in biologically relevant modules. Ulcerative colitis–specific SNPs were enriched in distal colon–related modules, suggesting that disease-specific mechanisms may result from altered lncRNA expression. The function of the IBD-associated SNP-adjacent lncRNA *IRF1-AS1* was explored in human myeloid cells, and our results suggested *IRF1-AS1* promoted optimal production of TNF-α, IL-6, and IL-23. A CRISPR/Cas9-mediated activation screen in THP-1 cells revealed several lncRNAs that modulated LPS-induced TNF-α responses. Overall, this study uncovered the expression patterns of lncRNAs in IBD that identify functional, disease-relevant lncRNAs.

## Abstract 2

<p>Gene correlation network analysis connects&nbsp;lncRNAs to biological pathways associated with more severe disease and genetic markers of inflammatory bowel disease.</p>

## Graphical abstract

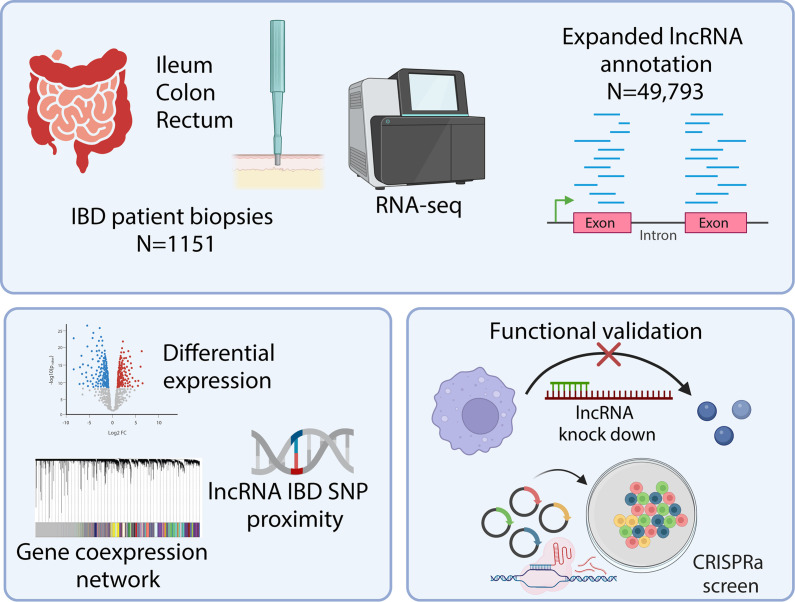

## Introduction

Ulcerative colitis (UC) and Crohn’s disease (CD) represent heterogenous gastrointestinal disorders and the predominant forms of inflammatory bowel disease (IBD) (1). Over 70% of genetic associations in IBD occur in the noncoding regions of the genome, with only a fraction appearing in coding genes. However, the genome is widely transcribed outside the coding space, and noncoding RNA transcripts are produced in intergenic regions, at enhancers, and bidirectionally from promoters (2–5). One class of noncoding RNA, long noncoding RNAs (lncRNAs), have received renewed interest as next-generation sequencing technologies have facilitated their widespread identification in the transcriptome. Nonetheless, the characterization of lncRNA expression in disease is far from complete owing to differences in lncRNA annotation across reference genome sets and lncRNA databases and the limited molecular tools available for lncRNA characterization (6).

Discrepancies and incomplete annotations have hindered the full transcriptomic and functional interrogation of lncRNAs. However, emerging evidence shows lncRNAs are effector molecules regulating diverse areas of biology including differentiation, cancer development, and gene regulation in inflammatory diseases (7–9). The function of lncRNAs is difficult to predict through sequence alone as few conserved functional domains have been characterized (10). However, the coregulation of lncRNAs with various biological pathways and their frequent cell type–specific expression suggests that coordinately expressed protein-coding genes may serve some utility in predicting lncRNA function. Accordingly, methods of coexpression analysis such as weighted gene correlation network analysis (WGCNA) have been used in large transcriptomic data sets to capture patterns of coexpressed genes in biological pathways or genes expressed in a cell type–specific manner. Moreover, the lower expression of lncRNAs compared with protein-coding genes adds additional challenges to rigorous statistical testing of lncRNA expression in large data sets. Low expression leads to increased percentage of zeros, necessitating the use of zero-inflated statistical models. The translation of recent advances in lncRNAs toward clinical application in IBD have been hindered by a lack of harmonized lncRNA annotation across data sets. We thus set out to comprehensively characterize lncRNA expression by applying a unified lncRNA annotation set to a large clinical IBD cohort. After controlling for potential confounding variables of the cohort, these analyses revealed expression of select lncRNA transcripts distinguishing disease states, severity, and affected regions. Using WGCNA, we uncovered putative functional roles for many lncRNAs and correlated gene modules to clinical features, biological pathways, and cell signatures. The lncRNA *IRF1-AS1* was identified as a hub gene in a module associated with inflammation and disease severity, and subsequent functional studies indicated that *IRF1-AS1* promoted inflammatory cytokine production by human myeloid cells. Finally, a CRISPR/Cas9-mediated activation (CRISPRa) screen in LPS-stimulated THP-1 cells identified other lncRNAs that appeared to modulate the production of TNF-α in myeloid cells.

## Results

### Quantification of gut biopsy transcriptomes.

**Figure 1:**
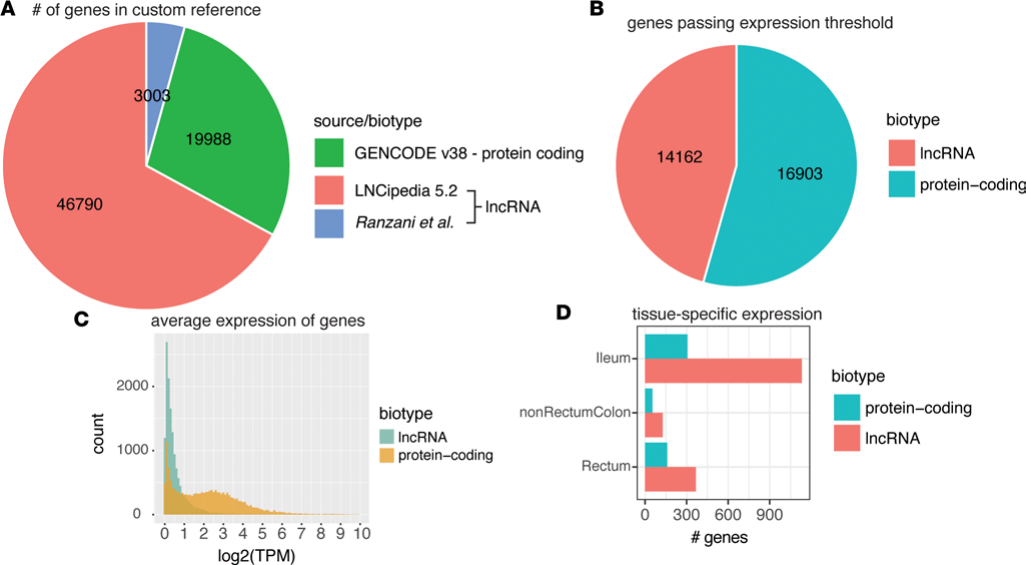
Summary of custom gene annotation for comprehensive lncRNA quantification and characterization of gene biotypes in the Mount Sinai Crohn’s and Colitis Registry data set. (**A**) The number of lncRNA and protein-coding genes and their corresponding reference source used for gene and transcript annotation. Protein-coding genes were isolated from GENCODE v38 while lncRNA genes were taken from the LNCipedia high-confidence set v5.2 and combined with the de novo–assembled lncRNAs transcripts identified in Ranzani et al. (12). (**B**) The number of lncRNA and protein-coding genes passing the expression threshold in the Mount Sinai Crohn’s and Colitis Registry (MSCCR) biopsy data set. (**C**) The log_2_-transformed, TPM-normalized expression levels of all expressed lncRNA and protein-coding genes averaged across all biopsy samples. (**D**) Tissue-specific expression of lncRNA and protein-coding genes as defined by containing at least 20 sequencing reads in at least 30% of samples in that tissue group but not in other tissue groups.

For quantification of lncRNA expression from RNA sequencing (RNA-Seq), we expanded the 18,805 lncRNA genes in GENCODE by using the lncRNAs from LNCipedia, a specialized, curated database of 46,790 lncRNA genes compiled from reference annotations such as Ensembl and Refseq, other lncRNA databases such as NONCODE, and several lncRNA-focused studies (11). We further supplemented the collection of lncRNAs from LNCipedia by adding 3,003 de novo–assembled lncRNAs from sorted lymphocyte transcriptomes from Ranzani et al. (12) for a total of 49,793 lncRNAs while retaining the 19,988 protein-coding genes in GENCODE (Figure 1A). This custom gene annotation set enabled comprehensive evaluation of protein-coding gene and lncRNA expression in gut biopsy transcriptomes from the Mount Sinai Crohn’s and Colitis Registry (MSCCR) (13, 14). We considered genes to be expressed if more than 10 sequencing reads in at least 40% of the libraries per disease classification (CD, UC, and healthy control [HC]) were quantified. After thresholding we transcriptome per million–normalized (TPM-normalized) the libraries using the remaining genes. With this threshold we found that only 28% of lncRNA genes were expressed in tissue biopsies compared with 90% of protein-coding genes (Figure 1B). The expression levels of protein-coding genes were higher than the expression of lncRNAs, but there was greater tissue specificity for lncRNAs compared with protein-coding genes (Figure 1, C and D). These results are consistent with prior observations that lncRNAs are generally expressed at lower levels but can be more tissue specific than protein-coding genes (15).

### Statistical models for lncRNA detection.

Differential gene expression analysis using standard methods from R packages such as DESeq2 or edgeR were not applied in this study because of a more complex data structure than these methods expect. In addition, the high percentage of zeros distorts model estimation and reduces power (16). Therefore, more appropriate alternatives were necessary, and zero-inflated generalized mixed-effects linear models were chosen instead (17). Clinical variables including demographics, comorbidities, and laboratory results of participants were explored to better model lncRNA expression and detect differentially expressed lncRNAs in UC and CD versus HCs. The samples were generally well balanced in terms of age, sex, race, ethnicity, IBD disease history, comorbidities, and medications (Table 1). Study participants with incomplete data records were removed, leaving 1,151 patients in the analysis data set. Sample collection varied based on tissue location, and we catered our model selection accordingly (see Methods). To ensure that differences in the demographics of the HCs and patients with IBD did not bias our results, we ran another set of models adjusting for demographics such as age, sex, race, ethnicity, and comorbidities. The overlap between the unadjusted and adjusted models was generally over 90% (Supplemental Table 1; supplemental material available online with this article; https://doi.org/10.1172/jci.insight.168988DS1), and only the genes significant in both the unadjusted and adjusted models were retained.

**Figure 2:**
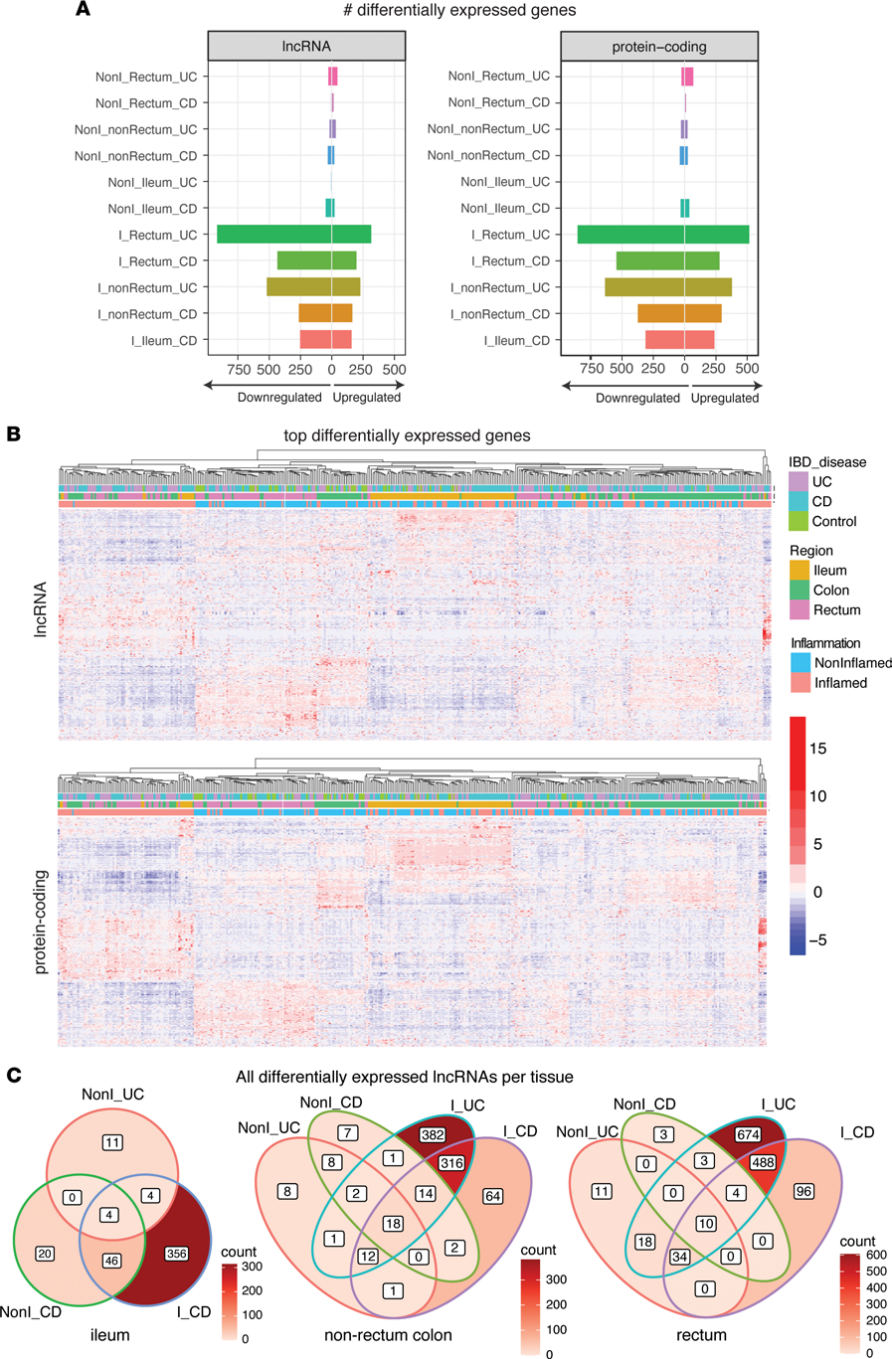
Differential expression analysis of the MSCCR biopsy data set. (**A**) Number of differentially expressed lncRNA and protein-coding genes. Bar plots depict number of significantly upregulated and downregulated genes [adjusted *P* < 0.05, absolute(log_2_fold-change) > 1] in each condition. (**B**) Heatmap showing expression of differentially expressed lncRNA [abs(log_2_fold-change > 1.5] and protein-coding genes for a random sampling of 200 inflamed samples and 200 noninflamed samples. Samples were clustered using the expression of both lncRNA and protein-coding genes and then separated into 2 heatmaps while keeping the sample order the same. (**C**) Overlap of significant lncRNAs [adjusted *P* < 0.05, abs(log_2_fold-change) > 1.5] for all comparisons in each tissue. NonI, noninflamed; I, inflamed; UC, ulcerative colitis; CD, Crohn’s disease.

This analysis revealed extensive differential expression of both lncRNAs and protein-coding genes in IBD, with 1,807 and 1,741 differentially expressed genes versus in HCs, respectively (Figure 2A and Supplemental Table 1). Most differentially expressed genes were seen in inflamed conditions, with few changes seen for either protein-coding genes or lncRNAs in the absence of inflammation (Figure 2A). For examining the overlap of differentially expressed genes identified in each condition, the samples were clustered using the genes with higher fold-change differences versus HCs to reduce the number of differentially expressed genes for plotting to 662 lncRNAs and 755 protein-coding genes. Heatmaps of the expression profile revealed that samples were most distinct based on tissue location and inflammation status, with contributions from both lncRNA and protein-coding genes (Figure 2B). Meanwhile, expression profiles for noninflamed tissue from HCs and patients with IBD were not as distinct (Figure 2B). For all 3 tissues, inflammation was the major driver of differential gene expression, with a large overlap between UC and CD suggesting shared regulatory processes (Figure 2C). These results show that lncRNAs demonstrate tissue specificity but also have pronounced changes of expression with inflammatory disease and that the full extent of lncRNA involvement is revealed with improved annotation.

### Construction of weighted coexpression networks.

**Figure 3:**
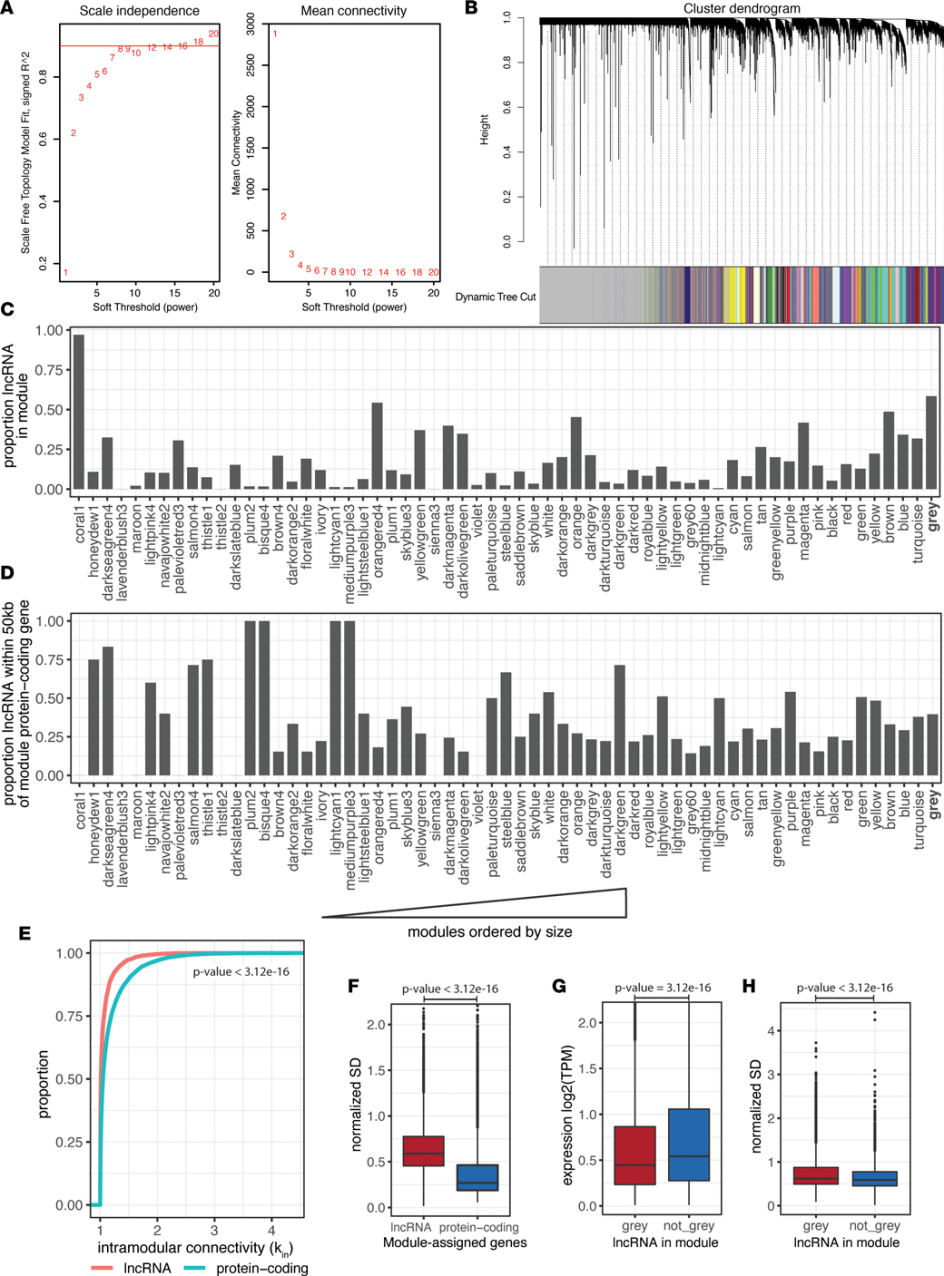
Construction and characterization of WGCNA network. (**A**) Soft-threshold selection process for scale independence of the data set. (**B**) Interaction of coexpressed genes based on a Topological Overlap Matrix (TOM) dissimilarity and the resulting cluster dendrogram. Each color represents one coexpression module, and branches above represent genes. (**C**) The proportion of lncRNAs in each coexpression module ordered by module size with smallest modules on the left. (**D**) Proportion of lncRNAs in each module within 50 kb of a protein-coding gene in the same module. Modules are ordered by size with smallest modules on the left. (**E**) The intramodular connectivity, i.e., connectivity of the gene to other genes within the same module, of lncRNA versus protein-coding genes. Differences in the empirical distribution function between the 2 curves were tested using a Kolmogorov-Smirnov test. (**F**) The normalized standard deviation between lncRNA and protein-coding genes in all modules except *gray*. The normalized standard deviation was calculated as the standard deviation of expression divided by the average expression of the gene. (**G**) The average log_2_(TPM) expression for lncRNAs in the *gray* module versus all nongray modules. (**H**) The normalized standard deviation between lncRNAs in the *gray* module versus all nongray modules. Box plots show the interquartile range (box), median (line), and minimum and maximum (whiskers). Differences in **F**–**H** were tested using a 2-sided *t* test.

We next sought to provide structure to the expression patterns of lncRNAs in IBD to reveal the underlying lncRNA network architecture. After regressing out relevant covariates, genes passing the expression threshold in 1,478 samples from inflamed and noninflamed biopsies from 855 patients with UC or CD and 343 noninflamed biopsies from 238 HCs were used to construct a coexpression network using WGCNA. Inflamed ileum samples were collected in CD, but few were collected in UC; therefore, ileum samples were omitted from the analysis. A soft-threshold power was used in the network to increase scale independence and modulate the mean connectivity of the network. A soft threshold of 8 was used to obtain an approximate scale-free topology of over 80% with saturation of scale independence at higher values (Figure 3A). Genes with similar expression profiles were clustered and divided into 60 modules after dynamic tree cutting of the cluster dendrogram (Figure 3B). Per WGCNA, colors were used for module identities. We analyzed module composition by quantifying the proportion of lncRNAs and comparing them by relative module size. Out of the 60 modules, 57 modules contained fewer than 50% lncRNAs (Figure 3C). Intriguingly, the *coral1* module was found to be composed of over 95% lncRNAs, suggesting a lncRNA-dominant regulatory hub (Figure 3C). There was a weak but insignificant trend toward higher lncRNA incorporation in larger modules, and lncRNAs in smaller modules were more often found in proximity (within 50 kB) of module protein-coding genes compared with larger modules (Figure 3, C and D). The degree of gene connectivity inside each module, defined as intramodular connectivity, was then calculated for each gene. Protein-coding genes had higher intramodular connectivity than lncRNAs (Figure 3E), suggesting a higher degree of synchronized expression for protein-coding genes compared with lncRNAs. To measure intersample variability of module-assigned lncRNAs and protein-coding genes, the standard deviation of each gene was calculated and normalized to the mean expression level. The normalized standard deviation revealed that lncRNAs had higher expression variability between samples than protein-coding genes (Figure 3F). Genes that could not be assigned to any module were placed in the *gray* module and 57% were lncRNAs. Unassigned lncRNAs had lower mean expression compared with module-assigned lncRNAs but similar standard deviation, indicating low expression may reduce detectable coexpression patterns (Figure 3, G and H). These results suggest higher regulatory variability of lncRNAs than protein-coding genes and lower network connectivity. Despite the variability, the results also indicate coregulation of gene expression in disease can occur through the concert of protein-coding and lncRNA genes.

### Module analysis and module-trait correlation.

**Figure 4:**
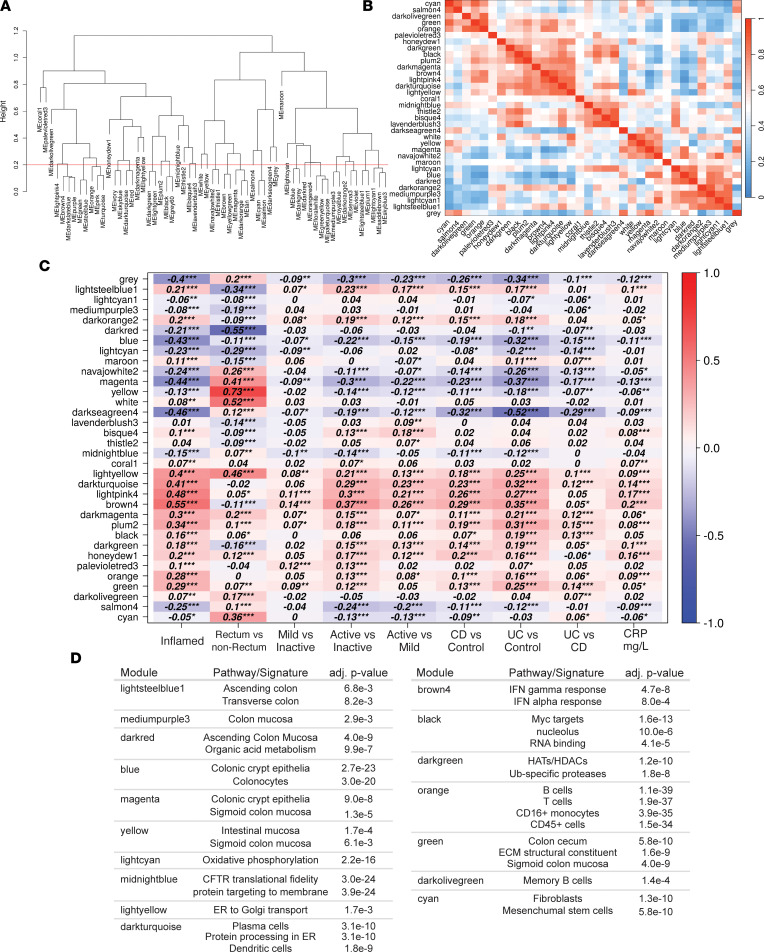
WGCNA module analysis and module-trait correlation. (**A**) An eigengene dendrogram identified groups of highly similar modules. Cluster groups that fell below the merging threshold of 0.2 were merged. (**B**) Eigengene adjacency heatmap of merged clusters identifies groups of correlated modules after merging. (**C**) Heatmap of the correlation between merged modules and clinical traits including biopsy inflammation status, rectal versus nonrectal colon sampling, mild versus inactive disease status, active versus inactive disease status, active versus mild disease status, CD versus control, UC versus control, UC versus CD, and C-reactive protein (CRP) levels. Each column corresponds to a clinical trait, and each row corresponds to a module. Each cell contains the correlation coefficient as calculated by Pearson correlation, and the *P* values are denoted by the number of stars. *P < 0.05, **P < 0.01, ***P < 0.001. (**D**) Enrichment of pathways for modules as determined by SaddleSum enrichment testing.

To further characterize the 60 modules, the module eigengenes were calculated. Module eigengenes are the first principal component of each module summarizing the module expression profile using dimensionality reduction. Clustering of module eigengenes revealed high similarity between some modules; therefore, similar modules were merged by cutting the module similarity dendrogram, resulting in 34 modules (Figure 4A). The eigengene adjacency heatmap showing the correlation between merged module eigengenes indicated broader patterns of correlated modules (Figure 4B). However, pathway analysis using the larger groups of correlated clusters failed to identify significantly enriched pathways. This suggests coregulated genes of specific pathways are organized in smaller modules and function together to carry out broader biological processes. Therefore, the merged set of 34 modules was used for further analyses (Supplemental Table 2). Intriguingly, the lncRNA-dominant *coral1* module remained distinct from other modules after merging.

Having demonstrated the replicability of the WGCNA modules on this data set, we returned to the network generated by analysis of the composite data set to examine whether identified modules correlated with basic clinical features. This module-trait association was calculated by correlating module eigengenes with clinical traits (Figure 4C). The traits included biopsy inflammation status, biopsy location, comparisons of IBD disease severity, disease designation, and CRP levels. IBD disease severity was determined for each biopsy at the time of sampling and stratified as inactive, mild, moderate, and severe disease. To improve sample number and reduce the number of comparisons, the moderate and severe IBD biopsy classifications were combined into a single “active” disease category, and categorical variables were binarized for correlation analysis. For the active versus inactive IBD trait, the *brown4*, *lightpink4*, and *darkturquoise* modules were the most positively correlated (*r* = 0.37, *r* = 0.3, and *r* = 0.29, respectively). The *brown4*, *lightpink4*, and *darkturquoise* modules were also correlated with inflammation status, CRP level, and the active versus mild IBD trait, suggesting that these modules are associated with disease severity and active disease processes, and were therefore identified as key modules for IBD activity. Additional associations were identified for biopsy location. The *yellow*, *white*, and *lightyellow* modules were most correlated for distal colon expression (*r* = 0.73, *r* = 0.52, and *r* = 0.46, respectively). However, the *yellow* and *lightyellow* modules diverged in their association with disease severity, with yellow being negatively and lightyellow being positively correlated with disease severity.

The division of coexpressed genes into modules is thought to reflect coregulation of genes acting in the same biological pathway. Since the function of most lncRNAs is unknown, we sought to annotate the lncRNAs in each module using pathway analysis to provide clues to their functional roles. Using the term enrichment analysis method, SaddleSum (18), we pulled the top 5 predicted pathways for each module from a selection of Gene Ontology Terms, Tissue and Cell Expression Barcodes, Reactome, and Molecular Signatures Database Hallmark Gene Sets. The top terms are presented in Supplemental Table 3 and Figure 4D. The pathway enrichment results demonstrated 16 modules had significant pathway enrichments while no significant enrichments were identified for 18 modules. Intestinal cell– and intestinal tissue–related signatures were the most abundant module enrichments (*yellow*, *blue*, *darkred*, *cyan*, *magenta*, *green*, *lightsteelblue1*, and *mediumpurple3*). Immune cell–related signatures were identified for modules *orange*, *darkturquoise*, and *darkolivegreen*. The *orange* module was composed of signatures for B cells, T cells, and monocytes; correlated well with the inflammation status trait (*r* = 0.28); and positively correlated with disease severity, suggesting this module captured inflammatory immune cell infiltrate. Reactome and Kyoto Encyclopedia of Genes and Genomes signatures were enriched for IFN response (*brown4*), endoplasmic reticulum (ER) to Golgi transport (*lightyellow*), histone acetylation/deacetylation (*dark* *green*), and oxidative phosphorylation (*lightcyan*). These results demonstrated that coexpressed genes of the colon in IBD can be divided into modules that are significantly enriched for genes of pathways that play roles in tissue homeostasis, cell regulation, and inflammatory processes.

**Figure 5:**
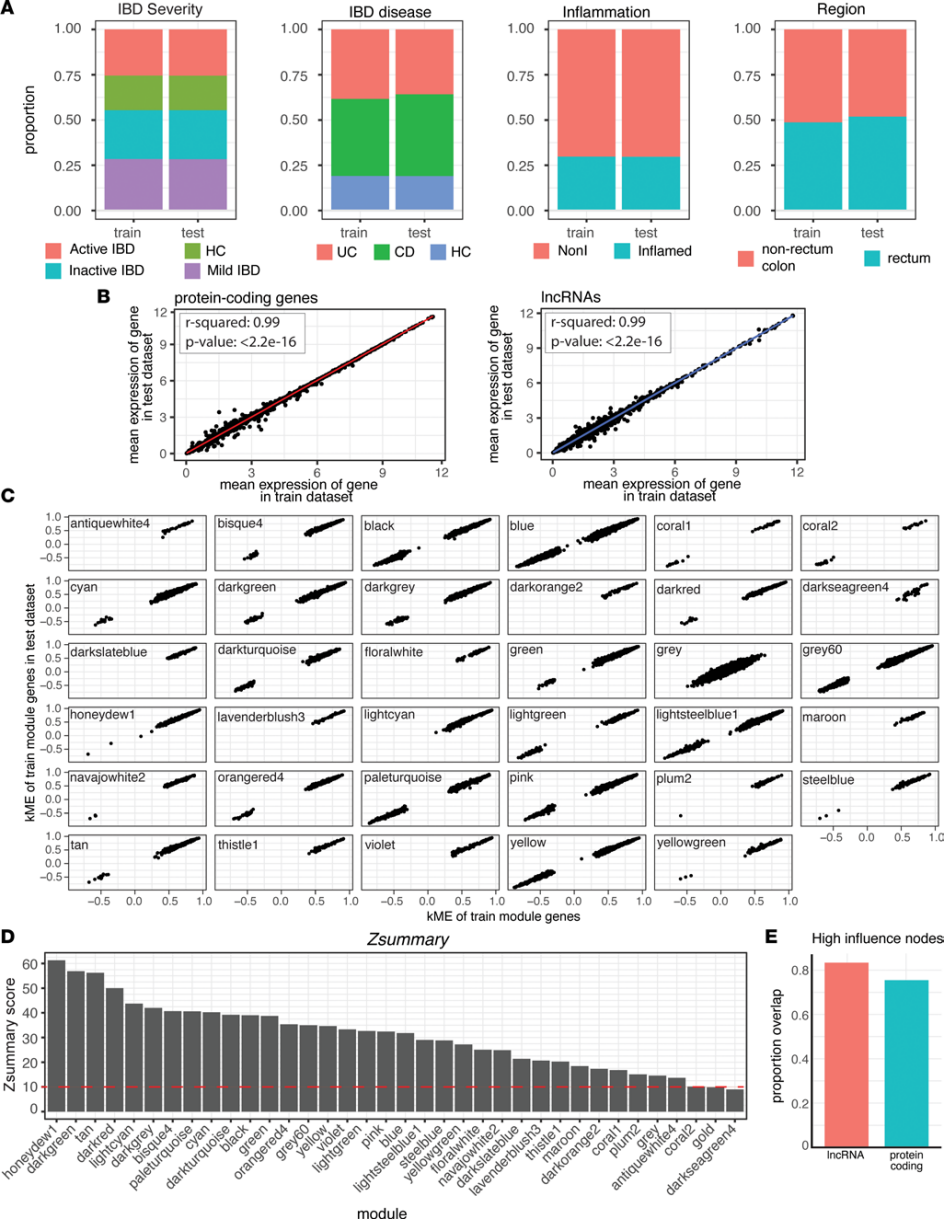
Network reproducibility between train and test data sets. (**A**) The distribution of samples across IBD disease severity, IBD disease category, inflammation status, and biopsy region after splitting the data set into a training set and a test set using stratified sampling to evenly separate based on IBD disease severity. (**B**) The average expression [log_2_(TPM + 1)] of protein-coding genes and lncRNAs in the train data set compared with the test data set and the results of fitting a linear regression. (**C**) The kME of module genes in the train data set and the kME of train data set module genes using the test data set. (**D**) The preservation of modules between networks calculated using the *Zsummary* statistic. (**E**) The proportion overlap of high-influence nodes from the top quantile of BC scores between train and test data sets.

Next, we wanted to ensure the modules produced by WGCNA on this data set were robust and replicable. Thus, the data set was split into a training set comprising 70% of the samples and a test set comprising the remaining 30% of the samples. We used a stratified sampling strategy to evenly distribute samples based on disease severity between the training and test set (Figure 5A). This sampling also resulted in equal distributions of biopsy sampling location, inflammation status, and IBD disease type (Figure 5A). There was a high correlation in the average expression of each gene between the 2 sets, suggesting the samples were well distributed for both lncRNAs and protein-coding genes (Figure 5B). We performed WGCNA as before to obtain a set of modules from the training set for use in comparison with the test set. The module membership values (kME), the correlation of the expression profile of a gene with the module eigengene, were then calculated for each gene. The expression profile of genes in the test set had similar kME as the genes in the training set when tested against training set module eigengenes (Figure 5C). Having determined that genes from the test set had analogous membership in training set modules, we then performed WGCNA on the test set to compare module composition between the 2 networks. A module preservation analysis was performed using the training set as the reference where a *Z* summary (*Zsummary*) statistic was calculated to measure the preservation of modules between networks (19). *Zsummary* scores > 10 are considered highly preserved while *Zsummary* scores < 5 are considered to have low preservation. Of the 34 modules in the test set, 32 had *Zsummary* scores of above 10, indicating that most modules were highly preserved between networks constructed from the 2 independent data sets (Figure 5D). Finally, a topological analysis was carried out on lncRNAs and protein-coding genes by calculating the betweenness centrality (BC) using the R package Igraph. In graph theory, BC measures the influence of a node over information flow in a module, and we used it to infer gene position in networks from each data set. High-influence nodes were determined by taking the top quantile of BC scores in the networks of both the train and test data sets. There was an overlap of 83% for lncRNAs and 75% for protein-coding genes for these high-influence nodes, suggesting similar topological layout between the train and test data sets (Figure 5E).

### Module visualization and identification of hub genes.

**Figure 6:**
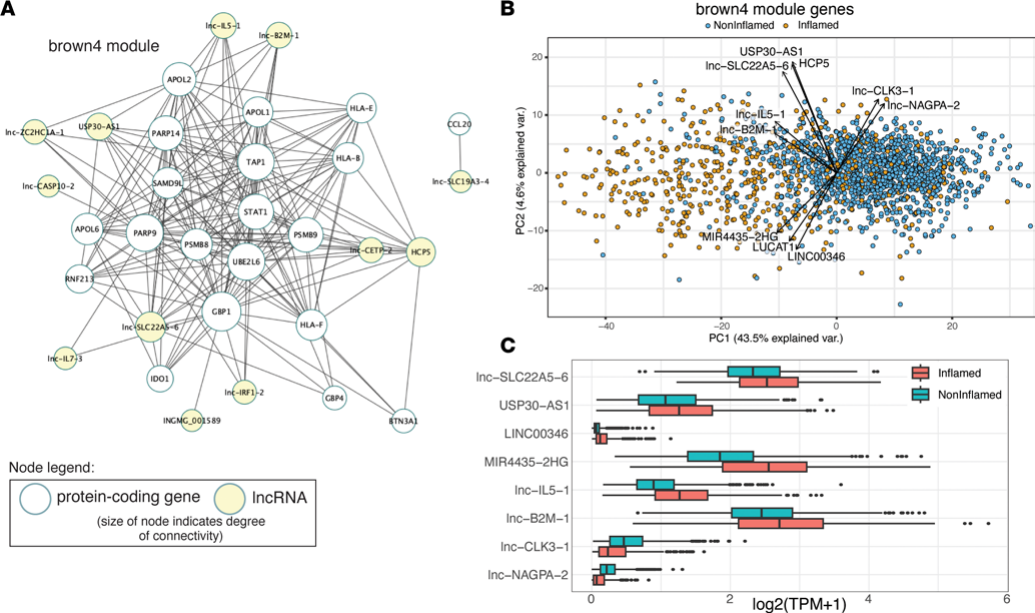
Hub genes and dimensionality reduction for the inflammation-associated *brown4* module. (**A**) The *brown4* coexpression module from the WGCNA was filtered with a connectivity threshold of 0.01 and exported for visualization into Cytoscape. The module network was filtered to show lncRNAs (yellow nodes) and their nearest connected neighbors, and the degree of connectivity between nodes was calculated and used to adjust the node size. (**B**) The expression of *brown4* module genes was isolated, principal component analysis was conducted, and the first 2 principal components (PC1 and PC2) were depicted. For *brown4* lncRNAs, the weights and direction of contributions were calculated as the hypotenuse between PC1 and PC2 loadings. Only the top lncRNA contributors to PC1 and PC2 are shown. (**C**) The log-normalized expression of the top lncRNA contributors between inflamed and noninflamed samples from the MSCCR biopsy data set. Box plots show the interquartile range (box), median (line), and minimum and maximum (whiskers).

To view the network structure of modules, we used an intramodular-connectivity threshold to limit modules of interest to top connected genes, called hub genes. The module networks were then filtered to display only lncRNAs and their nearest connected neighbors. The resultant genes in the *brown4* module — the module most positively correlated with disease severity — largely formed a single coherent network around protein-coding genes corresponding to an IFN response signature (Figure 6A). The most highly connected hub gene for the *brown4* module was the protein-coding gene *GBP1*, a recently reported IFN-inducible receptor for LPS involved in caspase-4–mediated pyroptosis (20, 21). Other protein-coding hub genes were involved in IFN-stimulated pathways, including MHC presentation (*TAP1*, *HLA-E*, *HLA-B*), STAT signaling (*STAT1*), and posttranslational ADP ribosylation modifications (*PARP9*, *PARP14*). Some of the protein-coding hub genes (*HLA-B*, *STAT1*) have been previously implicated in IBD susceptibility (22, 23). The top lncRNA hub gene, *lnc-SLC22A5-6*, also known as *IRF1-AS1*, has been associated with the IBD susceptibility loci rs17622378 and rs17622378 (24). Overall, the structure of the *brown4* network involved a similar degree of connectivity between protein-coding genes and lncRNAs. To identify putative lncRNA driver genes in the *brown4* module, a principal component analysis was conducted on the samples using the expression of the *brown4* module genes. Samples were best separated by inflammation status, consistent with the module-trait correlation analysis identifying this module as positively associated with inflammation. We calculated the contributions of lncRNAs to the first 2 principal components (PCs) and retained those with the highest contributions, finding 4 groupings of lncRNAs (Figure 6, B and C). The first set of lncRNAs were increased in inflammation and included *lnc-SLC22A5-6* and *USP30-AS1*; the second group was also increased in inflammation and included lncRNAs such as *LUCAT1*, *LINC00346*, and *MIR4435-2HG*; and the third group included the lncRNAs *lnc-IL5-1* and *lnc-B2M-1*. These first 3 groups had similar direction in the first PC but were delineated by the second PC. Finally, the fourth group of lncRNAs was higher in noninflamed samples and included lncRNAs such as *lnc-CLK3-1* and *lnc-NAGP1-2*. The higher contributions of these lncRNAs to *brown4* module expression suggested the possibility that these lncRNAs play roles in regulating the IFN response (Supplemental Table 3). Indeed, we have previously shown that *LUCAT1* is involved in the negative regulation of IFN responses by altering the splicing and stability of the nuclear receptor NR4A2, a downstream mediator of TNF-α signaling (25–27).

**Figure 7:**
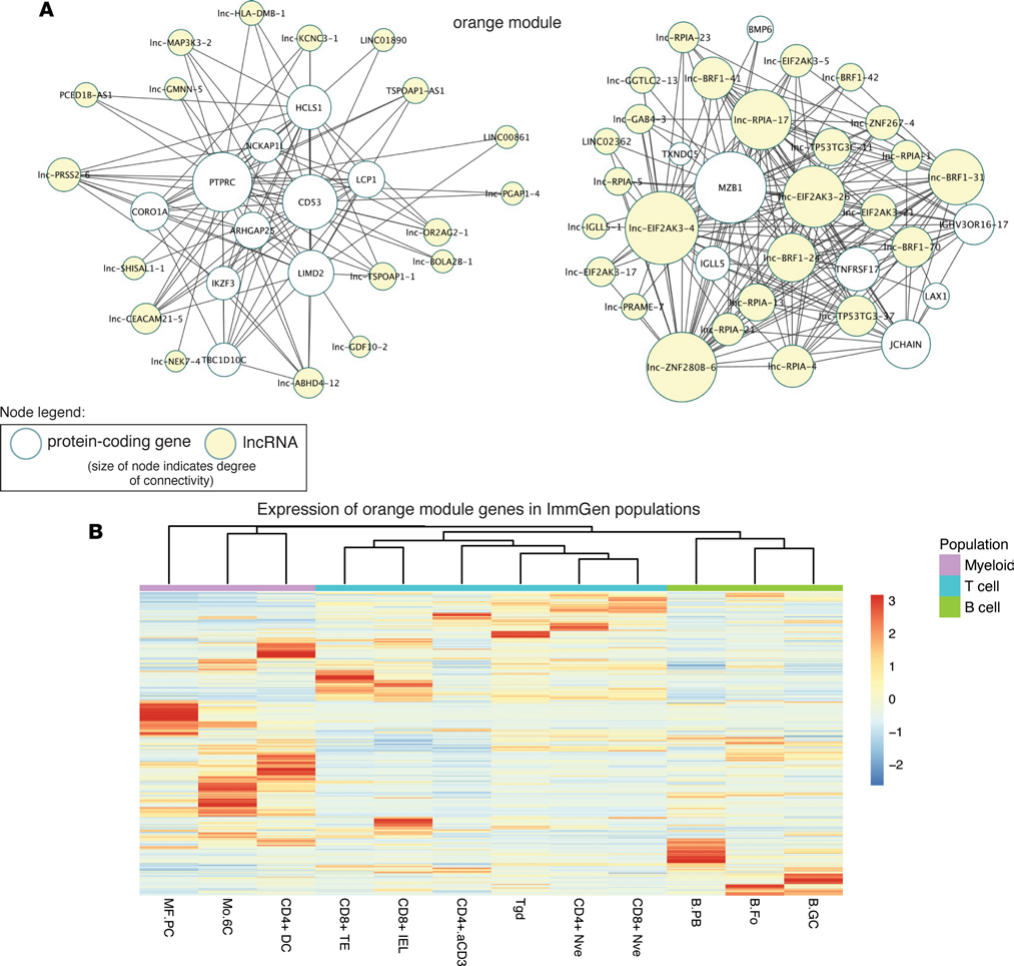
Visualization of hub genes for the immune cell signature–enriched *orange* module and expression of *orange* module genes in immune cell populations. (**A**) A connectivity threshold of 0.17 was applied to the *orange* module and exported for visualization into Cytoscape. The module network was filtered to show lncRNAs (yellow nodes) and their nearest connected neighbors, and the degree of connectivity between nodes was calculated and used to adjust the node size. (**B**) The expression of *orange* module genes was determined in various T, B, dendritic, and myeloid cell populations using ImmGen data. MF.PC, peritoneal macrophages; Mo.6C, Ly6C^+^ and Ly6C^–^ monocytes; CD4+ DC, CD4^+^ dendritic cells; CD8+ TE, CD8^+^ terminal effectors; CD8+ IEL, CD8^+^ intraepithelial lymphocytes; CD4+aCD3, CD4^+^ T cells activated with anti-CD3 and CD40; Tgd, γδ T cells; CD4+ Nve, CD4^+^ naive T cells; CD8+ Nve, CD8^+^ naive T cells; B.PB, B cell plasmablasts; B.Fo, follicular B cells; B.GC, germinal center B cells.

IBD pathology is driven by multiple immune cell types, such as myeloid, B, and T cells, and module analysis revealed the *orange* module as being enriched for multiple leukocyte cell signatures and increased with inflammation (Figure 4, C and D). To examine the organization of immune cell signatures in the *orange* module, the top connected lncRNAs and their nearest connected neighbors were visualized as a network. This network was found to be separated into 2 subnetworks (Figure 7A). The hub gene of one of the subnetworks was the protein tyrosine phosphatase CD45 (encoded by *PTRPC*), which was consistent with the leukocyte-centric cell signatures identified for the *orange* module (Figure 4D and Supplemental Table 3). The hub genes of another subnetwork have roles in efficient antibody production and plasma cell homeostasis, including immunoglobulin (Ig) heavy chain V genes. *MZB1* encodes a B cell–specific cochaperone of Grp94 active in ER stress conditions during Ig biosynthesis (28). *JCHAIN* encodes a polypeptide incorporated into IgA and pentameric IgM antibodies. Furthermore, *TNFRSF17* codes for B cell maturation antigen that promotes differentiation and survival of plasma cells (29). There was a high degree of connectivity for lncRNAs in this apparent plasma cell subnetwork, outranking even the protein-coding genes in some cases. Examination of the genomic location of the highly connected lncRNAs *lnc-EIF2AK3-4* and *lnc-ZNF280B-6* revealed they were within the Ig κ and λ loci, respectively. Antibody-secreting B cell populations have recently been implicated in IBD pathogenesis (30). To further explore the relationship of the *orange* module to immune cell populations, the expression of *orange* module genes was calculated from sorted immune cell transcriptomes from ImmGen (31). Subsets of *orange* module genes were specifically expressed in various cell types of myeloid, B, and T cell populations (Figure 7B). These results suggest that the *orange* module is composed of gene signatures from diverse immune cell types, with antibody-secreting B cells forming a distinct subnetwork highly enriched for several lncRNA hub genes.

### Relationship of modules to IBD genetics.

**Figure 8:**
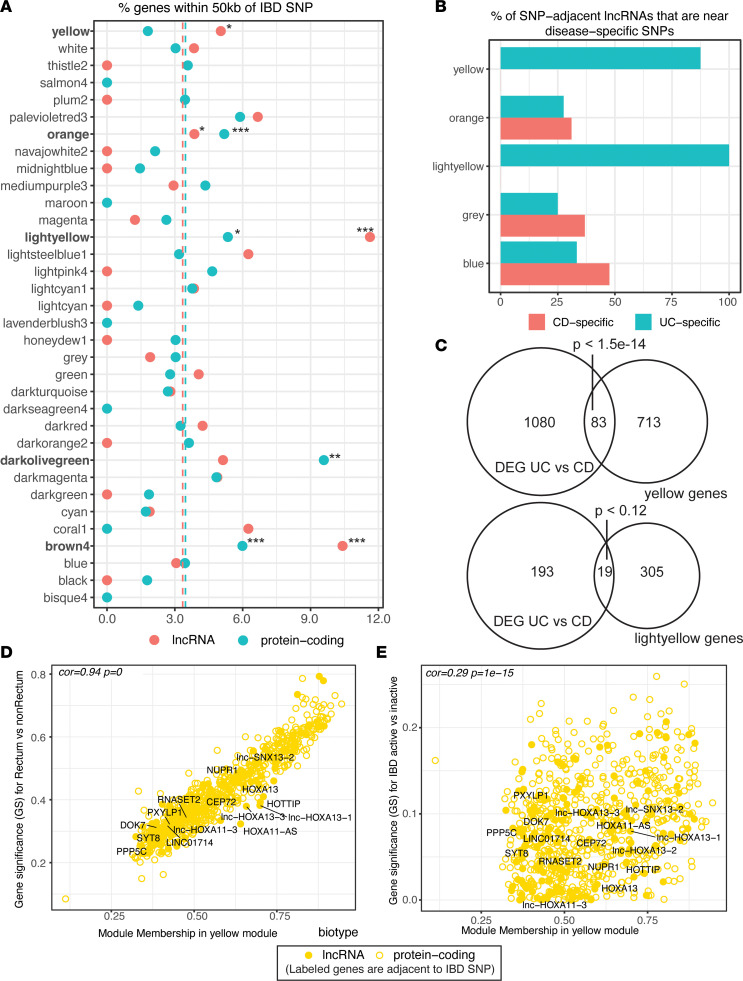
Relationship of modules to IBD genetics. (**A**) The percentage of SNP-adjacent lncRNA genes and protein-coding genes (within 50 kb) was calculated for each module (filled circles). Significance was determined using a Fisher’s exact test. The average percentage of SNP-adjacent lncRNA genes and protein-coding genes for all modules was also calculated (dashed lines). *P ≤ 0.05, **P ≤ 0.01, ***P ≤ 0.001. (**B**) The proportion of disease-specific (i.e., significant in one but not the other) SNP-adjacent lncRNAs was calculated for modules having at least 3 UC- or CD-specific SNP-adjacent lncRNAs. (**C**) Differential gene expression analysis was performed on UC versus CD samples in the rectum and nonrectum colon. All significant genes with adjusted *P* value below 0.01 and absolute log_2_ fold-change of 1 were intersected with the genes in the *yellow* and *lightyellow* modules. Significance testing of the overlap between the 2 gene sets was performed using a hypergeometric distribution with all genes included in the WGCNA as the total gene set. (**D**) For *yellow* module genes, the gene significance of each *yellow* module gene to the rectum versus nonrectum samples was calculated and plotted against the module membership of each *yellow* module gene. (**E**) For *yellow* module genes, the gene significance of each gene to active versus inactive IBD status was calculated and plotted against the module membership of each *yellow* module gene. For **D** and **E**, significance was calculated using Pearson correlation.

The altered lncRNA expression in the transcriptome reflects either cause or consequence of the disease state. To examine whether any modules harbor genetic susceptibility to IBD, we examined the proportion of protein-coding genes and lncRNAs that were within 50 kb of IBD risk loci identified by genome-wide association studies (GWAS). Across all genes in the network analysis, lncRNAs and protein-coding genes were equally likely to be adjacent to IBD-associated single nucleotide polymorphisms (SNPs) (Figure 8A, dashed lines). However, the enrichment of SNP-adjacent lncRNA and protein-coding genes was altered when looking across the 34 modules. Significance of enrichment was tested by bootstrapping size-matched random sets of genes. The disease severity–associated IFN response module *brown4* (Figure 4C) had significant enrichment for IBD SNP adjacency for both lncRNAs and protein-coding genes, supporting a causal role for lncRNAs in IBD etiology in a pathway that likely exacerbates disease. The *darkolivegreen* module was enriched for SNP-adjacent protein-coding genes, was enriched for a B cell signature (Figure 4D and Supplemental Table 3), and included SNP-adjacent B cell genes such as *BANK1* (Supplemental Table 3), which has been associated with systemic lupus erythematosus (32). The *lightyellow* module also had enrichment of lncRNAs and protein-coding genes adjacent to IBD GWAS SNPs whereas the *yellow* module had significant enrichment only for SNP-adjacent lncRNAs. Pathway enrichment analysis of *lightyellow* module revealed an ER to Golgi trafficking signature (Figure 4D and Supplemental Table 3), a biological process that is key to epithelial barrier function (33). Thus, the enrichment of SNP-adjacent lncRNAs in select disease-associated modules suggests a role for lncRNA involvement in IBD development and further suggests the network analysis has identified disease-relevant modules.

The patterns of colonic involvement between UC and CD are distinct, with rectal inflammation characteristic of UC compared with the noncontiguous, pan-colonic involvement found in CD. Despite differences in disease manifestation, the genetic architecture of CD and UC is largely shared, suggesting that disease-specific mechanisms are driven at the fringes by UC- and CD-specific genetics (22, 34). The proportion of SNP-adjacent lncRNAs that were either UC or CD specific for each module was determined to examine the potential lncRNA contribution to disease-specific presentation. Surprisingly, 100% of the SNP-adjacent lncRNAs in *lightyellow* and 88% of the SNP-adjacent lncRNAs in *yellow* modules were found to be adjacent to UC-specific SNPs (Figure 8B). Both modules were correlated with rectal involvement (Figure 4C), suggesting they are distal colon-specific modules that may be impacted more in UC than in CD. Consistent with this hypothesis, *yellow* module genes, but not *lightyellow*, had significant overlap with genes differentially expressed in a comparison of UC versus CD in the rectum (Figure 8C). The *yellow* module was also negatively correlated to disease severity, but this was not restricted to UC (Figure 4C). Correlations between individual genes and the indicated trait for *yellow* module genes were calculated between the *yellow* module and rectal gene expression. This correlation was defined as the gene-trait significance. Additionally, the membership of each gene in the *yellow* module was determined. As expected, there was a high correlation between rectum expression and gene membership in the *yellow* module (Figure 8D). The same analysis was carried out for the disease severity trait, which again validated a correlation between the *yellow* module and disease severity (Figure 8E). Several *yellow* module genes were adjacent to a UC-specific SNP in the *HOXA* locus, including the lncRNAs *lnc-HOXA11-3*, *lnc-HOXA13-1*, *lnc-HOXA13-2*, *lnc-HOXA13-3*, *HOXA11-AS*, and *HOTTIP* as well as the protein-coding gene *HOXA13*. *HOXA13* and the lncRNA *HOTTIP* are coregulated in hepatocellular carcinoma, and the lncRNA *HOXA11-AS* has been implicated in regulating proliferation of several cancers, including gastric cancer (35). These results suggest that disease-specific mechanisms of pathogenesis may be the outcome of SNP-driven lncRNA contributions acting within coordinately expressed gene programs.

### IRF1-AS1 promotes myeloid inflammatory cytokine production.

**Figure 9:**
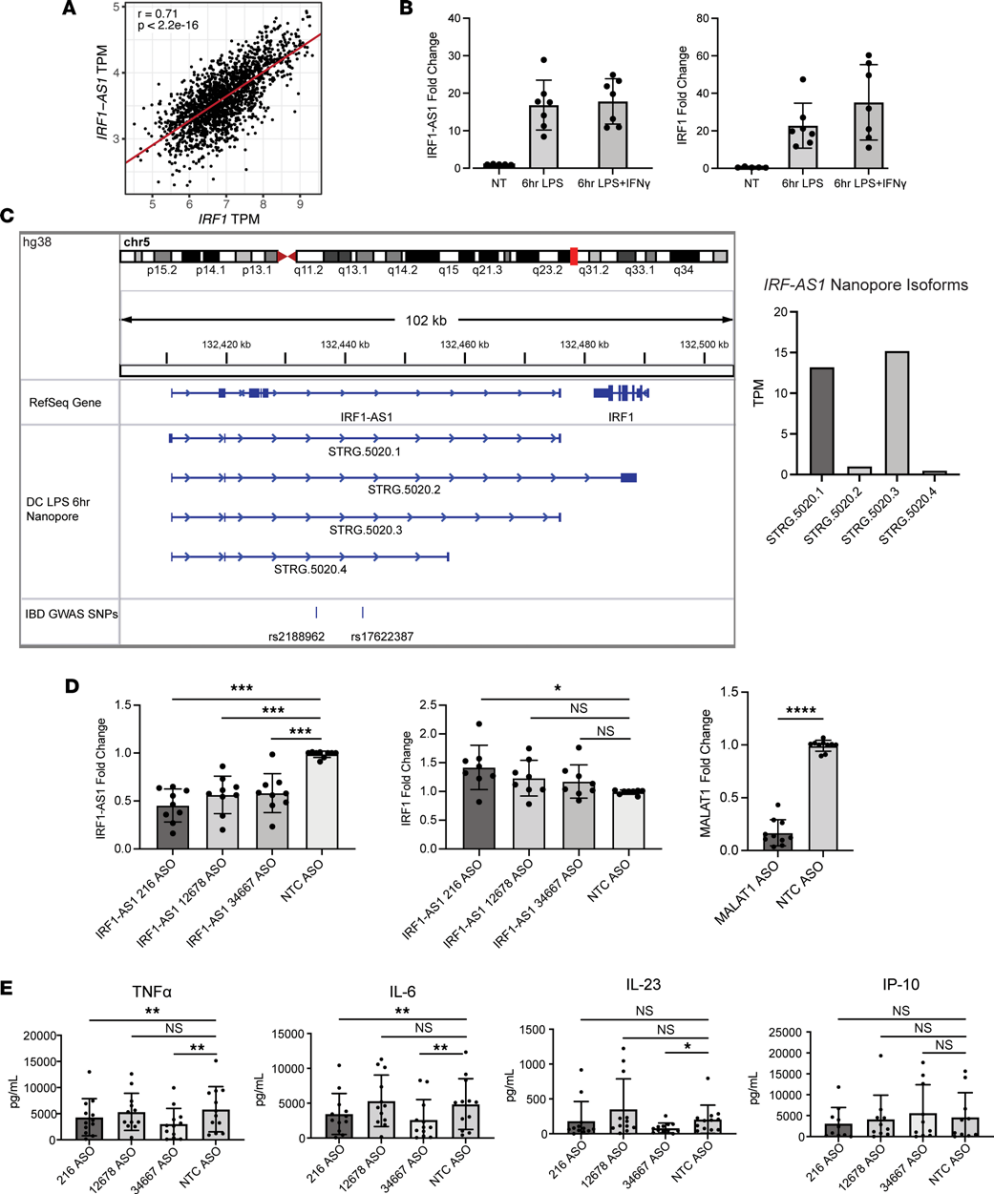
*IRF1-AS1* promotes inflammatory cytokine production. (**A**) Expression of *IRF1* and *IRF1-AS1* in colon samples of the MSCCR data set with calculated linear regression and Pearson correlation. (**B**) MDMs and MoDCs were stimulated with LPS or LPS + IFN-γ for 6 hours, and the expression of *IRF1-AS1* and *IRF1* was determined by quantitative PCR (qPCR) and compared with unstimulated samples. Data shown are from 2 experiments (1 for MDMs and 1 for MoDCs). (**C**) RNA extracted from MoDCs stimulated with LPS for 6 hours was converted to cDNA and sequenced with an Oxford Nanopore device. Stringtie of resulting sequences was used to determine the transcript sequences of *IRF1-AS1* (left). TPM levels were also calculated (right). (**D**) The transcript sequences of *IRF1-AS1* from Nanopore sequencing was used to design 3 highly specific ASOs (216, 12678, and 34667) along with *MALAT1* and NTC ASOs as controls. MDMs and MoDCs were differentiated from monocytes for 5 days before adding ASOs with RNAiMax for 48 hours. Cells were then stimulated for 6 hours with LPS before measuring *IRF1-AS1*, *IRF1*, and *MALAT1* expression by qPCR (*n* = 9, *n* = 8, and *n* = 10, respectively, across 3 experiments). (**E**) MDMs and MoDCs were differentiated from monocytes for 5 days before adding ASOs with RNAiMax for 48 hours. Cells were then stimulated for 6 hours with LPS before collecting supernatants from the cultures and measuring cytokine concentrations using Meso Scale Discovery multiplex cytokine ELISAs. Two experiments were performed on MDMs (*n* = 2 and *n* = 3), and 2 experiments were performed with MoDCs (*n* = 4 and *n* = 3); the results were combined for a total of 12 samples. Statistical significance for cytokine concentrations was determined using a 1-way ANOVA in GraphPad Prism. *P < 0.05, **P < 0.01, ***P < 0.001, ****P < 0.0001. Data represent mean ± SEM (**B**, **D**, and **E**).

To explore if any of the predicted lncRNAs identified in our analysis regulate an inflammatory pathogenesis seen in IBD, we focused our attention on the *brown4* module, which was enriched for an IFN response signature, correlated with inflammation and disease severity, and enriched for IBD GWAS SNPs (Figure 4, C and D; Figure 6, A and B; and Figure 8A). The lncRNA *lnc-SLC22A5-6*, also known as *IRF1-AS1*, was the highest connected lncRNA in *brown4* and overlapped with the IBD GWAS SNPs rs2188962 and rs17622378 (24). High correlation was observed between *IRF1-AS1* expression and the expression of the adjacent protein-coding gene *IRF1* (Figure 9A), a gene critical for the activation of myeloid cell defenses in response to IFN-γ and LPS (36). Thus, we hypothesized that *IRF1-AS1* plays a role in myeloid responses. To determine if *IRF1-AS1* functions to modulate myeloid cell responses, monocyte-derived macrophages (MDMs) and DCs (moDCs) were stimulated with LPS for 6 hours and *IRF1-AS1* transcription was measured. LPS stimulation increased both *IRF1-AS1* and *IRF1* transcription, but the addition of IFN-γ only further enhanced *IRF1* expression (Figure 9B). To identify the sequence of *IRF1-AS1* transcripts, we used Oxford Nanopore long-read sequencing on full-length cDNA isolated from LPS-stimulated moDCs after 6 hours. Multiple isoforms of *IRF1-AS1* were detected, but the most abundant isoforms did not overlap with *IRF1* (Figure 9C). Using these transcript sequences, we developed 3 antisense oligos (ASOs) to *IRF1-AS1* for reducing *IRF1-AS1* transcript levels and used RNAiMax to transfect the ASOs into MDMs and moDCs 48 hours before stimulation with LPS. Treatment with the ASOs reliably reduced *IRF1-AS1* levels by 50% compared with cells treated with a nontargeting control (NTC) ASO with knockdown of the *MALAT1* lncRNA using a *MALAT1* ASO, verifying transfection. Antisense lncRNAs often regulate the complementary protein-coding gene (37); therefore, *IRF1* levels in ASO-treated cells were also measured. However, minimal changes in *IRF1* levels were registered, and only with the 216 ASO, suggesting the lncRNA *IRF1-AS1* does not primarily regulate *IRF1* expression (Figure 9D). Cytokine production of cells was measured using multiplex cytokine enzyme-linked immunoassays (ELISAs) to determine if there were broader changes in the myeloid cell response to LPS with reduced *IRF1-AS1* expression. The ELISA results showed reduction of TNF-α, IL-6, and IL-23 protein levels in the supernatants of cells treated with the 216 ASO and the 34667 ASO, but not the 12678 ASO, compared with a NTC ASO whereas IFN-γ–induced protein 10 (IP-10) levels were not affected (Figure 9E). Therefore, expression of *IRF1-AS1* appears to promote inflammatory cytokine production in LPS-stimulated primary human myeloid cells.

### An integrative unbiased CRISPRa approach to identify lncRNAs that regulate inflammatory macrophage function.

While cell-specific expression and disease associations may indicate lncRNA involvement, a key challenge remains to unravel biological functions of lncRNAs. As lncRNA expression is often low, ASO/siRNA-knockdown approaches have proven challenging to effectively reduce expression in our hands. In addition, their noncoding nature renders CRISPR/Cas9-mediated small nucleotide deletions/insertions ineffective to affect function. To identify lncRNA function, we focused on inflammatory responses in macrophages, since they present a key cell population responsible for TNF-α production, a clinically validated therapeutic target in CD and UC. Specifically, we conducted a pooled CRISPRa screen using THP-1 dCas9-VP64/mCherry cells and targeted the transcription start sites (TSSs) of lncRNAs (6 sgRNAs per lncRNA) that were prioritized based on differential expression in gut biopsies of patients with IBD, associated with GWAS loci, or identified in our WGCNA. We further narrowed our screen to lncRNA transcripts readily detected in Oxford Nanopore long-read sequencing data of full-length cDNA isolated from LPS-stimulated moDCs, resulting in detection of 345 lncRNAs with 1,066 unique isoforms. The full-length sequence information obtained from Nanopore sequencing verified previously known TSS locations but also allowed detection of TSSs not previously annotated (Supplemental Table 4). In addition, we included positive (e.g., TNF-α, TLR4) and negative controls (intergenic sgRNAs that do not target any known lncRNA) to validate our screen.

**Figure 10:**
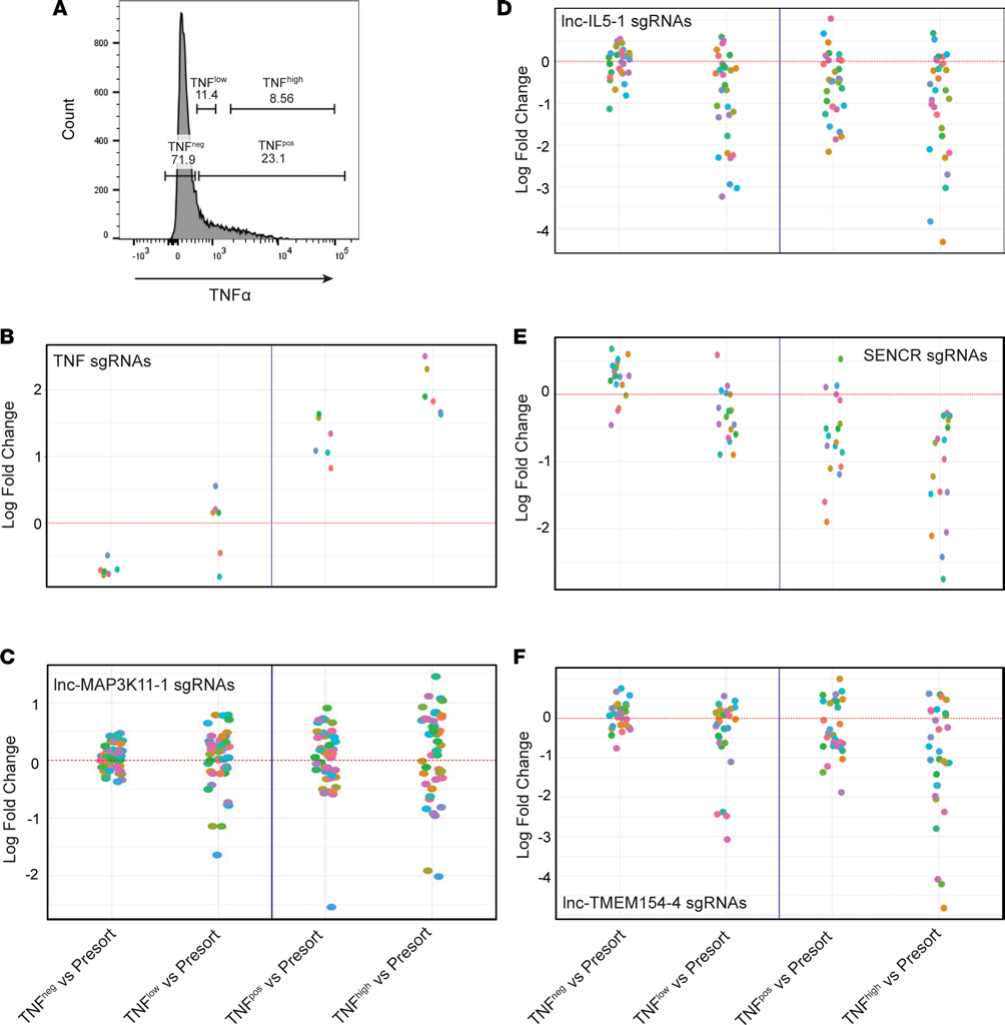
Identification of lncRNAs that modulate LPS-induced TNF-α responses using a CRISPRa screen. (**A**) THP-1 cells were stained for intracellular TNF-α and sorted into TNF-α^+^, TNF-α^–^, TNF-α^hi^, and TNF-α^lo^ populations. Enrichment and depletion of sgRNAs in TNF-α subpopulations were compared with presorted and TNF-α^–^ populations. (**B**) Log fold-change of sgRNAs targeting the TSS of *TNFA* reveal correlation with TNF-α levels, thus validating the CRISPRa screening potential. (**C**–**F**) Log fold-change of sgRNAs targeting lncRNAs that positively (*lnc-MAP3K11-1*, **C**) or negatively (*lnc-IL5-1*, **D**; *SENCR*, **E**; *lncTMEM154-4*, **F**) correlate with levels of TNF-α production.

Following transduction of THP-1 cells with the guide library, mature THP-1 cells were left untreated or stimulated with LPS in the presence of Brefeldin A. Subsequently, THP-1 cells were stained for intracellular TNF-α and sorted into TNF-α–positive (TNF^+^), TNF-α–negative (TNF^–^), TNF-α–high (TNF^hi^), and TNF-α–low (TNF^lo^) populations (Figure 10A and Supplemental Figure 1). Genomic DNA was analyzed by next-generation sequencing, and the relative abundance of each sgRNA was determined in each of the sorted TNF-α populations as well as pooled (presorted) cells. The log_2_ fold-changes in sgRNAs between the sorted TNF-α subpopulations and pooled cells or TNF^–^ cells were calculated using the STARS program from the Broad Institute (38). lncRNAs with STARS false discovery rate (FDR) < 0.25 were identified as potential lncRNAs that modulate (increase/decrease) LPS-induced TNF-α induction in macrophages.

Upon analyses, we identified 22 lncRNAs that exhibited enriched or depleted sgRNAs (FDR < 0.25) in 1 or more sorted TNF-α populations compared with presorted or TNF^–^ populations. Among these, *TNFA*, *TLR4*, and *IRF8* were the top hits exhibiting significant enrichment of sgRNAs in the TNF^hi^ population, thus validating our CRISPRa screen approach (Figure 10B and Supplemental Figure 2). In addition, we identified an enrichment of *lnc-MAP3K11-1* and *lnc-CEBPB-13* sgRNAs in TNF^+^ populations, suggesting that these lncRNAs promote LPS-induced TNF-α production in macrophages (Table 2 and Figure 10C). In contrast, we identified 9 lncRNAs that were negatively correlated with TNF-α production, showing a depletion of sgRNAs in TNF^+^ or TNF^hi^ population, suggesting they may suppress LPS-induced TNF-α production in macrophages (Table 2 and Figure 10, D–F). Among the top hits were *lnc-IL5-1* and *SENCR*, but also *LUCAT1* was identified to correlate with reduced TNF-α production. The latter observation is consistent with our previous observations that *LUCAT1* serves an antiinflammatory function by regulating NF-κB responses (26).

Overall, our CRISPRa screen successfully identifies a number of candidate lncRNAs that positively/negatively regulate inflammatory responses in macrophages that may be relevant to IBD development.

## Discussion

In the present study, RNA-Seq library quantification and analysis were performed on an expanded set of lncRNA annotations from gut biopsies in a large IBD cohort. This research resulted in an expression matrix of 19,899 lncRNAs and 16,903 protein-coding genes passing our detection threshold. Many new lncRNAs, especially those not included in GENCODE, were found to be differentially expressed in disease and inflammation and potentially play roles in IBD pathogenesis. WGCNA of the expressed genes revealed relationships of coexpression between lncRNAs and protein-coding mRNAs organized into modules of coordinate expression. Using the protein-coding genes in the modules for functional pathway enrichment, the results of this study implicate new key regulators and pathways in IBD for lncRNAs and protein-coding genes alike.

Much of the resolution from this study derives from the large sample size of the cohort data set. WGCNA is a systems biology approach particularly suited for large data sets for uncovering molecular pathways in disease, and the use of WGCNA for lncRNA research in inflammatory disease is on the rise (39–41). Since the ileum is a commonly affected tissue in CD, but not in UC, ileum samples were removed from the network analysis as not to bias the network toward the distinct transcriptional profile of ileum-based CD (42). A single network was constructed encompassing colon biopsies from both UC and CD, and 34 modules were associated with clinical features such as inflammation, location, or disease severity but were similarly associated between the 2 diseases. Although there are differences in UC and CD presentation, the findings using a unified network of colon biopsies from both diseases emphasized the similarities more than the differences. Therefore, the network was centered on cell signatures and pathways of the colon that were largely shared between the diseases. However, there were notable exceptions, including the *yellow* and *lightyellow* modules, which contained UC-specific SNPs and were biased toward expression in the rectum. Given the recent efforts to define the heterogeneity of IBD, subdividing samples based on disease type, anatomical location, or disease presentation could lead to higher resolution coexpression networks to identify context-specific modules and elucidate additional lncRNAs. Moreover, the greater variability and tissue specificity of lncRNA expression suggests that lncRNAs could be used in IBD to predict clinical outcomes or treatment response, or even used for subtype classification as has been done with lncRNAs in diseases such as breast cancer (43–45).

With the expanded lncRNA reference, a proximity-based GWAS SNP analysis was used to examine the enrichment of genetic susceptibility in our modules. The findings indicated increased enrichment of IBD SNP-adjacent lncRNAs in the disease severity–associated *brown4* and *lightsteelblue1* modules. The analyses were extended to find evidence of UC-specific SNP-adjacent lncRNAs in rectum-associated *yellow* and *lightyellow* modules. A closer examination of some of the SNP associations in these modules revealed multiple genes in the HOXA locus adjacent to a UC-specific SNP and including the protein-coding gene *HOXA13* and several lncRNAs. *HOXA13* is strongly expressed in the hindgut endoderm during early gut development with dosage effects on development of the epithelial layer of the rectum (46, 47), and lncRNAs in the HOXA are notably conserved among vertebrates (48). The consequence of disease-associated variants on lncRNAs in IBD is unknown, but a recent study on data from the Genotype Tissue Expression project has identified IBD-associated genetic variants associated with differences in lncRNA expression (49). Thus, future studies on the expression quantitative trait loci (eQTLs) of the impacted gene expression surrounding variants should be expanded to include a broad set of lncRNAs as was used in this study. However, noncoding variants identified by GWAS cannot readily be linked to a candidate causal gene, and therefore gene proximity-based methods are limited in establishing candidates. Additional studies that incorporate 3-dimensional genome conformation or colocalization with eQTLs to link SNPs with genes will be especially informative.

Due to the pervasive transcription of lncRNAs throughout the genome, it is unclear how many lncRNAs are merely byproducts of transcription and how many have functional roles. Through WGCNA, this study was able to provide putative functional annotation or associated cell types for IBD-expressed lncRNAs through “guilt by association,” many for the first time ever to our knowledge. Module-trait correlation analysis revealed the *brown4* module to be associated with disease severity and enriched for an IFN response signature. An IFN signature has been reported in blood and tissue for many autoimmune disorders, but the consequences of an elevated IFN signature in IBD have yet to be determined (50). Intriguingly, a coexpression analysis of an independent IBD cohort revealed a similar module enriched for an IFN signature with *STAT1* and *GBP1* as hub genes, and this signature was highly correlated with disease penetrance (51). Our results parallel this independent analysis while revealing additional information about lncRNA hub genes. Examination of the module network of *brown4* revealed the lncRNA *IRF1-AS1* was a highly connected hub gene and coexpressed with the neighboring *IRF1* gene. IRF1 is a transcription factor that regulates IFN responses in cells including myeloid cells. Knockdown of *IRF1-AS1* in LPS-stimulated myeloid cells reduced TNF-α, IL-6, and IL-23 production. However, expression of *IRF1* was minimally impacted after reduction of *IRF1-AS1* levels, and *IRF1* levels, but not *IRF1-AS1* levels, were increased by IFN-γ, suggesting *IRF1-AS1* regulates inflammatory cytokine protection without being directly regulated by IFN.

In addition to using hub genes to uncover functional lncRNAs, such as *IRF1-AS1*, an examination of the first 2 PCs of the *brown4* module revealed the lncRNA *LUCAT1* as a major contributor with elevated expression in inflamed biopsies. We have previously shown that *LUCAT1* regulates inflammation in myeloid cells by controlling the splicing of the transcription factor NR4A2 (26), suggesting that other lncRNAs identified using the PCs may also be functional in regulating inflammation. In addition to the functional lncRNAs identified through gene hubs or PCs, the lncRNA *HOXA11-AS* was adjacent to an IBD SNP and organized in the *yellow* module, a module highly associated with the rectum and enriched for UC-specific SNPs. We recently demonstrated the protective role of the highly conserved mouse ortholog *Hoxa11os* in colitis by restraining myeloid cell inflammatory cytokine production in the distal gut (52). Therefore, identifying lncRNAs adjacent to disease-associated SNPs also appears promising for identifying functionally relevant lncRNAs. Other studies have also investigated the role of lncRNAs in IBD by linking SNPs to nearby lncRNAs and functionally annotating the lncRNAs using the function of nearby protein-coding genes to find an enrichment of immune-regulatory processes (53, 54). Our study complements these efforts by expanding the functional annotation of lncRNAs using pathway enrichment analysis of the robust coexpressed modules obtained from our large IBD cohort. The use of different lncRNA annotations remains a barrier for cross-examining annotated lncRNA function across these various studies, and a formalized nomenclature like the one proposed by LNCipedia could help to alleviate this problem.

Despite the demonstrated utility of our gene coexpression networks, there are a few technical limitations to note. The transcriptomic data obtained for this study were based on a cross-sectional cohort whereas the expression of lncRNAs is likely dynamic. Future studies based on longitudinal data may reveal additional patterns of coexpression that could lead to a more complete understanding of lncRNAs in the pathogenesis of IBD. Moreover, the role of microRNAs, another noncoding RNA known to be regulated by lncRNAs, was not addressed in this study because our transcriptomic techniques were not suited to capture the microRNA compartment. Finally, the data set here consisted of unstranded RNA-Seq libraries, which presented difficulties in quantifying the expression of antisense lncRNAs. Pseudoalignment methods (as used in this study) have been investigated and found superior to alignment-based methods in quantifying lncRNA expression from unstranded libraries. One possible explanation for their superior performance is that the expectation maximization algorithm that pseudoalignment methods employ better models the random sampling of subsequences of spliced transcripts. Therefore, strand-specific methods of RNA-Seq library prep are recommended for further large-scale studies and clinical trials if lncRNA contribution to human disease is to be more fully understood as 32% of human lncRNAs are antisense to coding genes (55).

Addressing lncRNA functionality is challenging because of the limited molecular tools for characterizing lncRNA function. Despite reliably achieving *MALAT1* knockdown with the use of ASOs, we were only able to reduce *IRF1-AS1* levels by 50%. Nonetheless, using a CRISPRa screen in macrophages, we were able to identify lncRNAs that appear to modulate (increase/decrease) LPS-induced TNF-α responses. Among these was *LUCAT1*, verifying our previously reported antiinflammatory function for this lncRNA (26). In contrast, sgRNAs targeting *IRF1-AS1* did not significantly modulate LPS-induced TNF-α responses in our screen. This could be explained by the differences in approach (i.e., gain of function versus loss of function), with *IRF1-AS1* already reaching maximum functional capacity and a further increase having limited impact on modulation of TNF-α induction. In addition to *LUCAT1*, particularly *lnc-IL5-1* and *SENCR* show a strong negative correlation with the level of TNF-α production, suggesting that these lncRNAs may play important roles as negative regulators of macrophage inflammation. *SENCR* has been primarily associated with vascular disease and atherosclerosis, where it was reported to promote endothelial integrity by controlling the adherens junction through the RNA-binding protein CKAP4 and the membrane-bound CDH5 (56, 57). On the other hand, less is known about the tissue-specific expression, regulation, and potential functional role of *lnc-IL5-1*. Thus, the identification of these lncRNAs may uncover new and exciting biology, though confirmation and understanding the biology of each of these lncRNAs will require substantial efforts. Finally, the immunological context of our screen was limited to LPS-induced TNF-α production while we expect many of the lncRNAs screened may functionally impact other macrophage activation pathways, other immune cells such as T cells, or tissue epithelial cells. Future screening efforts may focus on understanding the functional role of these lncRNAs beyond macrophages.

In summary, an expanded set of lncRNA annotations was used to characterize lncRNA expression more fully in IBD, with a gene coexpression network analysis providing insights into the coregulation of lncRNA and protein-coding gene expression associated with clinical features and tissue and cell type specificity. We expect that our in silico prioritization of lncRNAs will provide a foundational resource for novel target identification, biomarker evaluation, and future functional studies of IBD-associated lncRNAs.

## Methods

Supplemental Methods are available online with this article.

### Data set information.

The data set used in this study was the MSCCR of biopsy whole-transcriptome sequencing data from a cross-sectional cohort composed of 1,170 patients enrolled in the MSCCR from December 2013 through September 2016 (for more details, see Supplemental Methods).

### Reference annotation.

The identification and annotation of noncoding genes are iterative and ongoing processes and multiple reference sets exist (58). For a uniform lncRNA annotation resource, the human lncRNA database LNCipedia was used (11). A custom reference annotation of nonredundant transcripts for both protein-coding genes and lncRNAs was constructed by combining protein-coding transcripts isolated from human transcriptome version GENCODE release 38 of GENCODE (GRCh38.p13) with the high-confidence set of lncRNAs from LNCipedia v 5.2. Additional lncRNAs resulting from de novo transcriptome assembly of human lymphocyte subsets from Ranzani et al. were included after lifting over to hg38 using the UCSC liftover tool (12, 59).

### Module visualization and identification of hub genes.

A connectivity threshold of 0.01 and 0.17 was applied to the *brown4* and *orange* modules, respectively, to reduce the number of edges in the final network. The module networks were exported and displayed in Cytoscape and filtered on lncRNAs and their nearest connected neighbors (60). The degree of connectivity of each node was calculated and used to adjust the node size. The expression of *brown4* module genes was then isolated, and principal component analysis was carried out in R using the *prcomp* function. For *brown4* lncRNAs, the weights and direction of contributions for top connected lncRNAs were calculated as the hypotenuse between PC1 and PC2 loadings. For the *orange* module genes, human gene symbols were converted to mouse gene symbols, and the heatmap of gene expression was plotted using the normalized set of RNA-Seq ImmGen data from National Center for Biotechnology Information Gene Expression Omnibus (NCBI GEO) data set GSE109125.

### SNP-based analysis.

IBD GWAS SNPs were collected from the NHGRI-EBI GWAS Catalog for Jostins et al., Liu et al., and de Lange et al. (22, 24, 61). Significant associations for UC, CD, and IBD were collected and separated into UC-specific, CD-specific, and IBD-shared SNPs. Genes were extended by 50 kb on each side and intersected with the genomic location of each SNP using bedtools version 2.30.0 (62). The number of lncRNAs and protein-coding genes within the 100 kb window at each SNP were tabulated for each module. Bootstrapping was performed to test the statistical enrichment of SNP-adjacent lncRNAs and protein-coding genes by sampling the same number of genes with the same proportion of lncRNAs and protein-coding genes and calculating the proportion of each adjacent to an SNP. This process was repeated 10,000 times for each module, and the actual value was compared to the resulting distribution to calculate the *P* value. The overlap of the *yellow* and *lightyellow* genes with the significant genes in the UC versus CD comparison was calculated, and a Fisher’s exact test was used to test for significance, with the number of protein-coding and lncRNA genes that passed the expression threshold for the WGCNA (23,083 genes) set as the background.

### Statistical modeling of differential expression.

Gene-level summarized RNA fragment counts were generated using the summarizeToGene function in tximeta (63) followed by TPM normalization. Tissue-specific lncRNA genes were defined as being above 0.5 TPM in at least 30% of the respective tissue but not in the other tissues. TPM counts were further log-transformed within each gene as the following:

  (Equation 1) 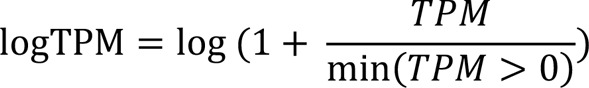.

The differential expression (DE) analyses were conducted separately for ileum, nonrectal colon, and rectum samples because of differences in sample collection. Ileum samples were collected from 659 patients, with 6 patients with CD having 1 inflamed and 1 noninflamed samples, and the remaining 653 patients having a single sample. Nonrectal colon samples were collected from 655 patients. Each patient had 3 inflamed and 3 noninflamed samples. Rectum samples were collected from 882 patients: 623 of the patients had a single noninflamed sample, 245 patients had a single inflamed sample, and 14 patients had 1 noninflamed and 1 inflamed sample. Samples from all 3 locations were sequenced in 125 batches.

DE analyses were done using zero-inflated mixed-effects Gaussian model (R function *NBZIMM:lme.zig*) (64). If the model did not converge, non–zero-inflated model was applied (R function *nlme:lme*) (65). Ileum and rectum samples were analyzed on sample level, with batch as a random-effects variable. Colon samples were analyzed on patient level with 3 inflamed and 3 noninflamed samples per patient and patient ID and batch included in the model as the random-effects variables. Gene expressions (logTPM) were modeled against the combined explanatory variable of disease status and the biopsy sample inflammation status as noninflamed controls, noninflamed UC, inflamed UC (except for ileum samples), noninflamed CD, and inflamed CD. The second set of models was used to adjust for the effects of the clinical variables: race, ethnicity, sex, and history of asthma, thyroid disease, rheumatoid arthritis, psoriasis, ankylosing spondylitis, osteopenia/osteoporosis, cancer, appendectomy, depression, and tobacco use. Gene expression was modeled in genes with statistically significant DE in inflamed UC and inflamed CD samples only against disease severity, duration, and medication class. All reported *P* values were FDR adjusted (66). The results were visualized in R using bar plots, heatmaps, and Venn diagrams.

### lncRNA CRISPRa library design.

An sgRNA CRISPRa library was designed and synthesized by the Broad Institute. The sgRNAs were designed to target the TSSs of 345 lncRNAs expressed in LPS-stimulated moDCs identified through Nanopore-sequenced cDNA from moDCs stimulated with LPS for 6 hours. Approximately 6 sgRNAs per TSS were designed. In addition, 680 negative control intergenic sgRNAs, which do not target any known lncRNA, were included. Finally, protein-coding genes *TLR4*, *MYD88*, *IRAK4*, *IRAK1*, *TRAF6*, *RELA*, *NFKB1*, and *TNFA* were targeted by 10 sgRNAs each as positive controls. The final library contained 5,837 total sgRNAs, which were cloned into lentiviral CRISPRa vector pXPR502 (Broad Institute).

### CRISPRa screen data analysis.

The log_2_ fold-changes between the sorted (based on TNF-α level) cells and pooled cells were calculated per sgRNA for THP-1 dCas9-VP64/mCherry cells (generated in-house from parent THP-1 cells [ATCC]) at day 21. Statistical analysis of the log_2_ fold-changes were conducted using the STARS program from the Broad Institute (38). The STARS program calculates a score using the probability mass function of a binomial distribution. The calculation was performed for all sgRNAs that ranked in the top 4% of the log_2_ fold-changes. For genes with at least 2 sgRNAs ranked in the top 4%, a score from the sgRNA with the lowest rank in the top 4% was assigned to the gene. *P* values and FDRs were calculated for each gene based on a null distribution generated by permutation of the original library (see Table 2). lncRNAs with STARS FDR < 0.25 were identified as candidates in which activation of the lncRNA would either increase or reduce the TNF-α expression in THP-1 cells at day 21.

### Statistics.

Statistical tests and *P* values are in the figure and table legends.

### Study approval.

The protocol was approved by the Icahn School of Medicine at Mount Sinai Institutional Review Board, and patients gave written informed consent (13, 14).

### Data availability.

The MSCCR of biopsy whole-transcriptome sequencing data are public on NCBI GEO GSE193677. Values for data points depicted in graphs and behind reported mean values in the manuscript and supplement are available in the Supporting Data Values file online.

## Author contributions

JLJ, AOH, KAF, JET, KH, and DS designed and supervised this study. JLJ, DS, and KH drafted the manuscript. DS performed the statistical analysis. JLJ, AH, and SA analyzed the data. AS, RK, HI, MSF, WMS, and CA were involved with initial data set collection and curation. EMN generated data. LE and YS performed the CRISPRa screen and analysis. JP, LSG, TV, and GB provided critical review of the research design and manuscript. All authors read and approved the final manuscript.

## Supplementary Material

Supplemental data

Supplemental table 1

Supplemental table 2

Supplemental table 3

Supplemental table 4

Supporting data values

## Figures and Tables

**Table 1:**
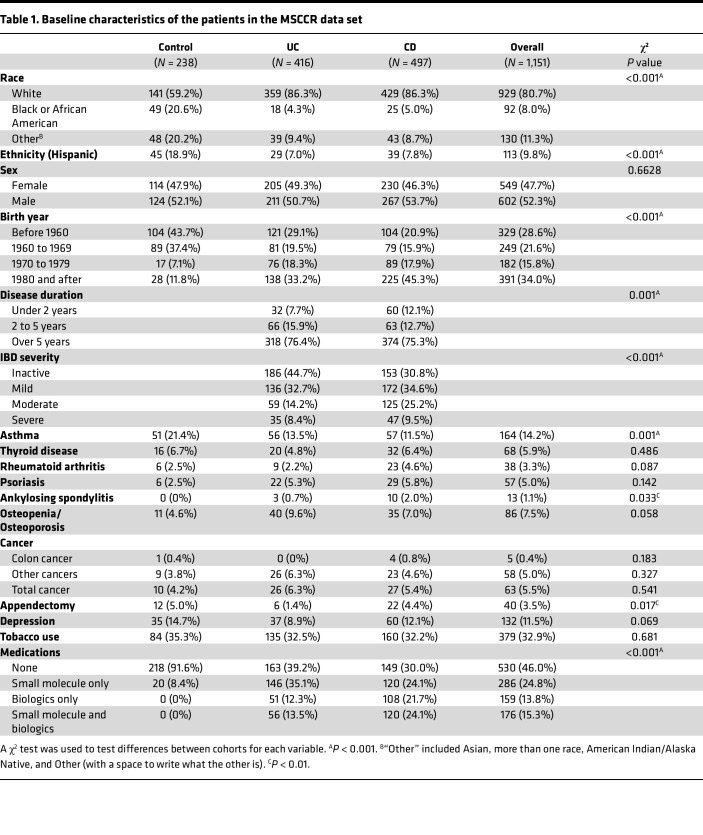
Baseline characteristics of the patients in the MSCCR data set

**Table 2:**
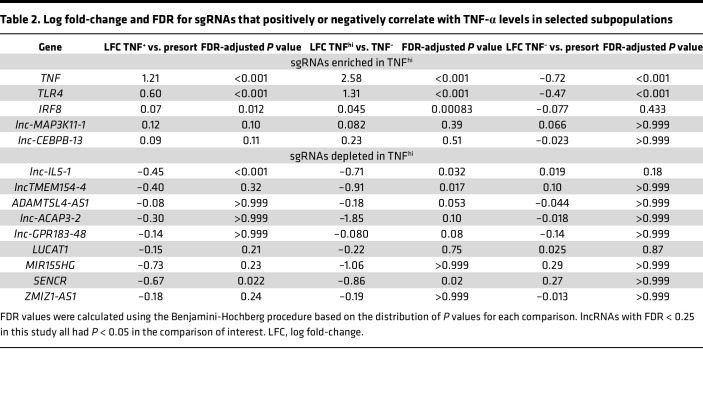
Log fold-change and FDR for sgRNAs that positively or negatively correlate with TNF-α levels in selected subpopulations

## Version 1 02/08/2024

Electronic publication

## References

[1] Sartor RB (1995). Current concepts of the etiology and pathogenesis of ulcerative colitis and Crohn’s disease. Gastroenterol Clin North Am.

[2] Bertone P (2004). Global identification of human transcribed sequences with genome tiling arrays. Science.

[3] Carninci P (2005). The transcriptional landscape of the mammalian genome. Science.

[4] Djebali S (2012). Landscape of transcription in human cells. Nature.

[5] Sigova AA (2013). Divergent transcription of long noncoding RNA/mRNA gene pairs in embryonic stem cells. Proc Natl Acad Sci U S A.

[6] Fritah S (2014). Databases for lncRNAs: a comparative evaluation of emerging tools. RNA.

[7] Fatica A, Bozzoni I (2014). Long non-coding RNAs: new players in cell differentiation and development. Nat Rev Genet.

[8] Schmitt AM, Chang HY (2016). Long noncoding RNAs in cancer pathways. Cancer Cell.

[9] Atianand MK (2017). Immunobiology of long noncoding RNAs. Annu Rev Immunol.

[10] Guo X (2015). Advances in long noncoding RNAs: identification, structure prediction and function annotation. Brief Funct Genomics.

[11] Volders P-J (2018). LNCipedia 5: towards a reference set of human long non-coding RNAs. Nucleic Acids Res.

[12] Ranzani V (2015). The long intergenic noncoding RNA landscape of human lymphocytes highlights the regulation of T cell differentiation by linc-MAF-4. Nat Immunol.

[13] Suárez-Fariñas M (2013). Intestinal inflammation modulates the expression of ACE2 and TMPRSS2 and potentially overlaps with the pathogenesis of SARS-CoV-2-related disease. Gastroenterology.

[14] Argmann C (2021). Molecular characterization of limited ulcerative colitis reveals novel biology and predictors of disease extension. Gastroenterology.

[15] Derrien T (2012). The GENCODE v7 catalog of human long noncoding RNAs: analysis of their gene structure, evolution, and expression. Genome Res.

[16] Li Q (2019). lncDIFF: a novel quasi-likelihood method for differential expression analysis of non-coding RNA. BMC Genomics.

[17] Ran D, Daye ZJ (2017). Gene expression variability and the analysis of large-scale RNA-seq studies with the MDSeq. Nucleic Acids Res.

[18] Stojmirović A, Yu Y-K (2010). Robust and accurate data enrichment statistics via distribution function of sum of weights. Bioinformatics.

[19] Langfelder P (2011). Is my network module preserved and reproducible?. PLoS Comput Biol.

[20] Santos JC (2020). Human GBP1 binds LPS to initiate assembly of a caspase-4 activating platform on cytosolic bacteria. Nat Commun.

[21] Wandel MP (2020). Guanylate-binding proteins convert cytosolic bacteria into caspase-4 signaling platforms. Nat Immunol.

[22] Jostins L (2012). Host-microbe interactions have shaped the genetic architecture of inflammatory bowel disease. Nature.

[23] Ahmad T (2006). Genetics of inflammatory bowel disease: the role of the HLA complex. World J Gastroenterol.

[24] Liu JZ (2015). Association analyses identify 38 susceptibility loci for inflammatory bowel disease and highlight shared genetic risk across populations. Nat Genet.

[25] Agarwal S (2020). The long non-coding RNA LUCAT1 is a negative feedback regulator of interferon responses in humans. Nat Commun.

[26] Vierbuchen T (2023). The lncRNA LUCAT1 is elevated in inflammatory disease and restrains inflammation by regulating the splicing and stability of NR4A2. Proc Natl Acad Sci U S A.

[27] Mix KS (2012). Orphan nuclear receptor NR4A2 induces synoviocyte proliferation, invasion, and matrix metalloproteinase 13 transcription. Arthritis Rheum.

[28] Rosenbaum M (2014). MZB1 is a GRP94 cochaperone that enables proper immunoglobulin heavy chain biosynthesis upon ER stress. Genes Dev.

[29] O’Connor BP (2004). BCMA is essential for the survival of long-lived bone marrow plasma cells. J Exp Med.

[30] Uzzan M (2022). Ulcerative colitis is characterized by a plasmablast-skewed humoral response associated with disease activity. Nat Med.

[31] Heng TSP (2008). The Immunological Genome Project: networks of gene expression in immune cells. Nat Immunol.

[32] Kozyrev SV (2008). Functional variants in the B-cell gene BANK1 are associated with systemic lupus erythematosus. Nat Genet.

[33] Graham DB (2016). TMEM258 is a component of the oligosaccharyltransferase complex controlling ER stress and intestinal inflammation. Cell Rep.

[34] Ventham NT (2013). Beyond gene discovery in inflammatory bowel disease: the emerging role of epigenetics. Gastroenterology.

[35] Quagliata L (2014). Long noncoding RNA HOTTIP/HOXA13 expression is associated with disease progression and predicts outcome in hepatocellular carcinoma patients. Hepatology.

[36] Kamijo R (1994). Requirement for transcription factor IRF-1 in NO synthase induction in macrophages. Science.

[37] Villegas VE, Zaphiropoulos PG (2015). Neighboring gene regulation by antisense long non-coding RNAs. Int J Mol Sci.

[38] Doench JG (2016). Optimized sgRNA design to maximize activity and minimize off-target effects of CRISPR-Cas9. Nat Biotechnol.

[39] Luo M (2021). Weighted gene coexpression network and experimental analyses identify lncRNA SPRR2C as a regulator of the IL-22-stimulated HaCaT cell phenotype through the miR-330/STAT1/S100A7 axis. Cell Death Dis.

[40] Rui Q (2021). Identification and validation of key long non-coding RNAs using co-expression network analysis in Crohn’s disease. Ann Palliat Med.

[41] Scicluna BP (2020). The leukocyte non-coding RNA landscape in critically ill patients with sepsis. Elife.

[42] Weiser M (2018). Molecular classification of Crohn’s disease reveals two clinically relevant subtypes. Gut.

[43] Milevskiy MJG (2016). Long-range regulators of the lncRNA HOTAIR enhance its prognostic potential in breast cancer. Hum Mol Genet.

[44] Bao S (2019). Computational identification of mutator-derived lncRNA signatures of genome instability for improving the clinical outcome of cancers: a case study in breast cancer. Brief Bioinform.

[45] Su X (2014). Comprehensive analysis of long non-coding RNAs in human breast cancer clinical subtypes. Oncotarget.

[46] Roberts DJ (1998). Epithelial-mesenchymal signaling during the regionalization of the chick gut. Development.

[47] Warot X (1997). Gene dosage-dependent effects of the Hoxa-13 and Hoxd-13 mutations on morphogenesis of the terminal parts of the digestive and urogenital tracts. Development.

[48] Degani N (2021). Highly conserved and cis-acting lncRNAs produced from paralogous regions in the center of HOXA and HOXB clusters in the endoderm lineage. PLoS Genet.

[49] De Goede OM (2021). Population-scale tissue transcriptomics maps long non-coding RNAs to complex disease. Cell.

[50] Andreou N-P (2020). Inflammatory bowel disease pathobiology: the role of the interferon signature. Ann Gastroenterol.

[51] Sudhakar P (2020). Understanding the molecular drivers of disease heterogeneity in Crohn’s disease using multi-omic data integration and network analysis. Inflamm Bowel Dis.

[52] Shmuel-Galia L (2023). The lncRNA HOXA11os regulates mitochondrial function in myeloid cells to maintain intestinal homeostasis. Cell Metab.

[53] Mirza AH (2015). Transcriptomic landscape of lncRNAs in inflammatory bowel disease. Genome Med.

[54] Yarani R (2018). The emerging role of lncRNAs in inflammatory bowel disease. Exp Mol Med.

[55] Ma L (2013). On the classification of long non-coding RNAs. RNA Biol.

[56] Lyu Q (2019). SENCR stabilizes vascular endothelial cell adherens junctions through interaction with CKAP4. Proc Natl Acad Sci U S A.

[57] Pierce JB, Feinberg MW (2020). Long noncoding RNAs in atherosclerosis and vascular injury: pathobiology, biomarkers, and targets for therapy. Arterioscler Thromb Vasc Biol.

[58] Frankish A (2020). GENCODE 2021. Nucleic Acids Res.

[59] Hinrichs AS (2006). The UCSC Genome Browser Database: update 2006. Nucleic Acids Res.

[60] Shannon P (2003). Cytoscape: a software environment for integrated models of biomolecular interaction networks. Genome Res.

[61] De Lange KM (2017). Genome-wide association study implicates immune activation of multiple integrin genes in inflammatory bowel disease. Nat Genet.

[62] Quinlan AR, Hall IM (2010). BEDTools: a flexible suite of utilities for comparing genomic features. Bioinformatics.

[63] Love MI (2020). Tximeta: reference sequence checksums for provenance identification in RNA-seq. PLoS Comput Biol.

[64] Zhang X, Yi N (2020). NBZIMM: negative binomial and zero-inflated mixed models, with application to microbiome/metagenomics data analysis. BMC Bioinformatics.

[66] Benjamini Y, Hochberg Y (1995). Controlling the false discovery rate: a practical and powerful approach to multiple testing. J R Stat Soc Series B Stat Methodol.

