# Supplementary material for: Gene coexpression networks reveal a broad role for lncRNAs in inflammatory bowel disease
Source: JCI Insight. 2024 Feb 8;9(3):e168988. doi: 10.1172/jci.insight.168988 (PMC10967393; doi:10.1172/jci.insight.168988)

Supplemental Material and Methods

Detailed Dataset Information

The MSCCR data consisted of two datasets. The *Gene expression dataset* contained RNA-seq counts of 69,692 protein-coding and lncRNA genes from our custom reference annotation from 2,515 samples collected from 1,170 patients and sequenced in 125 batches. After filtering out genes with low expression, 14,162 long non-coding and 16,903 protein coding RNAs remained in the analysis dataset. The *Clinical variables dataset* contained 800 variables divided into the following domains: demographics, medical history, medications, family history, Harvey-Bradshaw index (CD scoring system), colitis activity index (patient-reported UC scoring system), IBDSESCD (CD endoscopic scoring system), IBDUCEIS (UC endoscopic scoring system), IBDMESUC (UC Mayo endoscopic scoring system), extraintestinal manifestations, laboratory test results, CD diagnosis information, diagnosis criteria, UC diagnosis information (Montreal classification), dysplasia, intermediate colitis, surgical history of non-control patients, surgical history list, IBD MPDAI (Pouch index for patients with current ileo-anal pouch), IBDPHQ8 depression questionnaire, IBDPMIBDINDEX Manitoba questionnaire, and study eligibility. The following clinical variables were included in the analysis: IBD diagnosis (Control, UC, or CD), race (White, Black, Other), ethnicity (Non-Hispanic or Hispanic), age group (born before 1960, between 1960 and 1969, between 1970 and 1979, and 1980 or after), inflammation region (ileum, colon non-rectum, or rectum), sample type (non-inflamed or inflamed), and history of asthma, thyroid disease, rheumatoid arthritis, psoriasis, ankylosing spondylitis, osteopenia/osteoporosis, cancer, appendectomy, depression and tobacco use. 17 patients had incomplete records and were removed from the analysis. A small number of samples (5 inflamed control and 7 UC inflamed ileum samples) were removed from the analysis reducing the number of patients by 2 in the control group. The summary of the remaining 1,151 patients' baseline characteristics is presented in Table 1.

RNA-seq quantification

Alignment-based methods of gene quantification work poorly for unstranded RNA-seq libraries, especially for lncRNAs, but better performance can be achieved with pseudoalignment (60). Therefore, the pseudoalignment method provided by Salmon was used (61). *Fastq* files were trimmed using *cutadapt* using the options "-a AGATCGGAAGAGCACACGTCTGAACTCCAGTCA --minimum-length 1 -j 15" (62). The trimmed *fastq* files were pseudo-aligned and quantified using Salmon version 1.5.2. Salmon results files were imported into R using the package *tximeta* Bioconductor release 3.14 (63). Gene level counts were generated using the function *summarizeToGene* with the options "countsFromAbundance='lengthScaledTPM'". "Indeterminate" disease samples were removed. A gene expression threshold was establishing using the function *filterByExpr* in the *R* package *edgeR* version 3.32.1 with the options "min.prop = 0.3, min.count = 10" with the "group" option set as IBD disease (i.e., "UC", "CD", or "Control"). For WGCNA, gene level counts were generated using the function *summarizeToGene* with the options "countsFromAbundance='lengthScaledTPM'". Ileum samples and "Indeterminate" disease samples were removed. Genes with low expression were filtered with the function *filterByExpr* in the *R* package *edgeR* version 3.32.1 with the options "min.prop = 0.7, min.count = 20" with the "group" option set as IBD disease (i.e., "UC", "CD", or "Control") (64). The *R* package *limma-voom* was used to correct for the following covariates: RQN.RIN_phase1_and_phase2, Extraction_Batch_phase1_and_phase2_UPDATED, ExonicRate_Phase1andPhase2, rRNA_rate_phase1_and_phase2, Demographics_gender, and Study_Eligibility_age_at_endo and the covariate adjusted residuals were used for subsequent WGCNA analysis. For the tissue specificity analysis, the gene-level summarized gene counts were generated using the function summarizeToGene in *tximeta* followed by TPM-normalization. Tissue-specific lncRNAs and protein-coding genes were defined as having at least 20 read counts in at least 30% of the respective tissue but not in the other tissues.

WGCNA

WGCNA was used to identify transcriptomic networks using correlation patterns (65). Using the covariate-adjusted expression matrix of non-ileum samples, a soft threshold of 8 was selected to provide a degree of scale independence over 0.8 and unsigned networks were constructed. Subsequently, modules were called using dynamic branch cutting and a merging threshold of 0.20 was used to reduce 60 modules to 34 modules.

Pathway Enrichment Analysis

The genes in each module were extracted and pathway analysis was performed using SaddleSum (19). For each module, the gene connectivity (i.e., the row sum of the adjacency matrix) was used as the weights for SaddleSum. An adjusted p-value below 0.05 was set as the significance threshold. Databases of pathways that were queried included GO Terms, Tissue and Cell Expression Barcodes, Reactome, and MSigDb Hallmark Gene Sets (66-69).

Train and Test dataset comparison

The dataset used for WGCNA was separated into a training dataset comprising 70% of the samples and a test dataset comprising the remaining 30% of the samples. The samples were separated into these two groups using a stratified sampling strategy using the createDataPartition function in the R package *caret* to separate samples equally based on IBD disease severity. WGCNA was performed on the training dataset in the same manner as the combined dataset to identify 34 modules for use as the reference network. WGCNA was then also performed on the test dataset in the same manner to identify 36 modules. Module preservation between the two networks was evaluated in two ways: 1) the kME values of the training dataset modules were obtained before calculating the kME values of the test network using the module assignments from the reference network (73) and 2) calculating a statistic to evaluate preservation of module density and connectivity as outlined previously (18). The modules of each network were converted to an *Igraph* object using the function wgcna2igraph adapted from https://github.com/jtlovell/limmaDE2/. Nodes were filtered to have at least a kME of 0.05 and an adjacency of 0.1. A topological analysis was then carried out by calculating betweenness centrality scores on remaining nodes using the R package *Igraph*. The top 20% of scores for both protein-coding genes and lncRNAs was determined for each network and deemed high influence nodes and the overlap was determined to assess preservation of topology.

Culture of MDMs and MoDCs and knock-down of IRF1-AS1.

Monocytes (CD14+) were ordered from Biological Specialty Company and differentiated into MDMs using recombinant human GM-CSF (20ng/mL) or differentiated into MoDCs using a cocktail of recombinant human IL-4 (50ng/mL) and recombinant human GM-CSF (100ng/mL). After 5 days of differentiation, MDMs and MoDCs were stimulated with LPS (200ng/mL) or LPS (200ng/mL) and IFNγ (25ng/mL) to induce *IRF1-AS1* and *IRF1* expression which was detected using SYBR-green directed qPCR or TaqMan probe-based qPCR (Thermo Fisher Scientific). The sequence of the primers used to detect *IRF1-AS1* were `CCTGGGGCCGCAACC` for the forward primer and `GCTGGCCATCCTCAGGAC` for the reverse primer. To knockdown *IRF1-AS1* and *IRF1* transcripts, cells were plated at 0.5x10^6 cells per well in a 24 well plate. *IRF1-AS1* transcripts were knocked-down in differentiated MDMs and MoDCs using Affinity Plus gapmer anti-sense oligonucleotides purchased from Integrated DNA Technologies (IDT). The sequence for the *IRF1-AS1* 216 ASO was `CTGCAGAGGTGATCTA`, the sequence for the *IRF1-AS1* 12678 ASO was `AATGCGTTGCCCTACT`, and the sequence for the *IRF1-AS1* 34667 ASO was `AGATTTGCGCCCCTTA`. *MALAT1* and NTC ASOs were purchased as off-the-shelf controls from IDT. ASOs were reconstituted to a 100mM stock concentration and the ASO transfection mix was created by diluting RNAiMax (Thermo Fisher Scientific) 1:10 in Opti-MEM (Gibco) and then mixed with an equal volume of 1 mM ASO diluted with Opti-MEM. After 10 minutes of incubation, 50 µL of the ASO transfection mix was added to the well of a 24 well plate for a final concentration of 50 nM ASO and 5 µL RNAiMax per well. Cells were incubated with ASOs for a further 48 hours before being stimulated with LPS to induce cytokine production. The concentration of cytokines in the supernatant was detected using Meso Scale Discovery multi-plex cytokine ELISAs as directed by the manufacturer.

Pooled CRISPRa Screen

THP-1 dCas9-VP64/mCherry cells were transduced with the guide library at a representation of 500X library coverage in 3 replicates. Cells were transduced at an MOI between 0.3 and 0.5 to ensure that each cell received at most 1 sgRNA. Cells were spun in 12-well plates at 2000 rpm for 2 h at 30 °C. Cells were grown with 1µg/ml puromycin selection beginning at 24 h post-transduction. The cells were grown under selection until 50 million cells/replicate were available and then 50ng/ml PMA was added for 24

hours followed by fresh media for 24 hours. Cells were then treated with 1µg/ml LPS (Invivogen) and 10µg/ml Brefeldin A for 5 hours.

Cells were detached using Accutase, stained with LIVE/DEAD™ Fixable Aqua Dead Cell Stain (ThermoFisher) for 20 minutes, fixed with 2% PFA for 10 minutes, resuspended in PBS and stored at 4ºC overnight. The next day, samples were suspended in Cytofix/cytoperm buffer (BD Biosciences) for 20 minutes, washed twice with PermWash buffer (BD Biosciences), stained with Anti-Human TNFα (BD Biosciences 559321) in PermWash for 30 minutes at 4ºC, washed twice with PermWash and then resuspended in PBS. 5e6 cells were removed from each replicate as a pre-sort sample. Next, TNFα positive, TNFα negative, TNFα high, and TNFα low populations for each replicate were sorted into separate tubes using Aria (BD FACSAria IIu). Genomic DNA (gDNA) isolation was performed using the QIAamp DNA Blood Midi Kit or QIAamp DNA Blood Maxi Kit (Qiagen) and OneStep PCR Inhibitor Removal (Zymo). Purified gDNA was sent to the Broad Institute for NGS and deconvolution.

Supplemental Figure and Table legends

Supplementary Figure 1 – Flow Cytometry Plots of TNFα before or after sorting of subpopulations. (**A**) Intracellular staining of TNFα in unstimulated or after LPS stimulation in 3 replicate samples. (**B**) Distribution of TNFα in TNFα^{pos}, TNFα^{neg}, TNFα^{low} or TNFα^{high} populations after sorting.

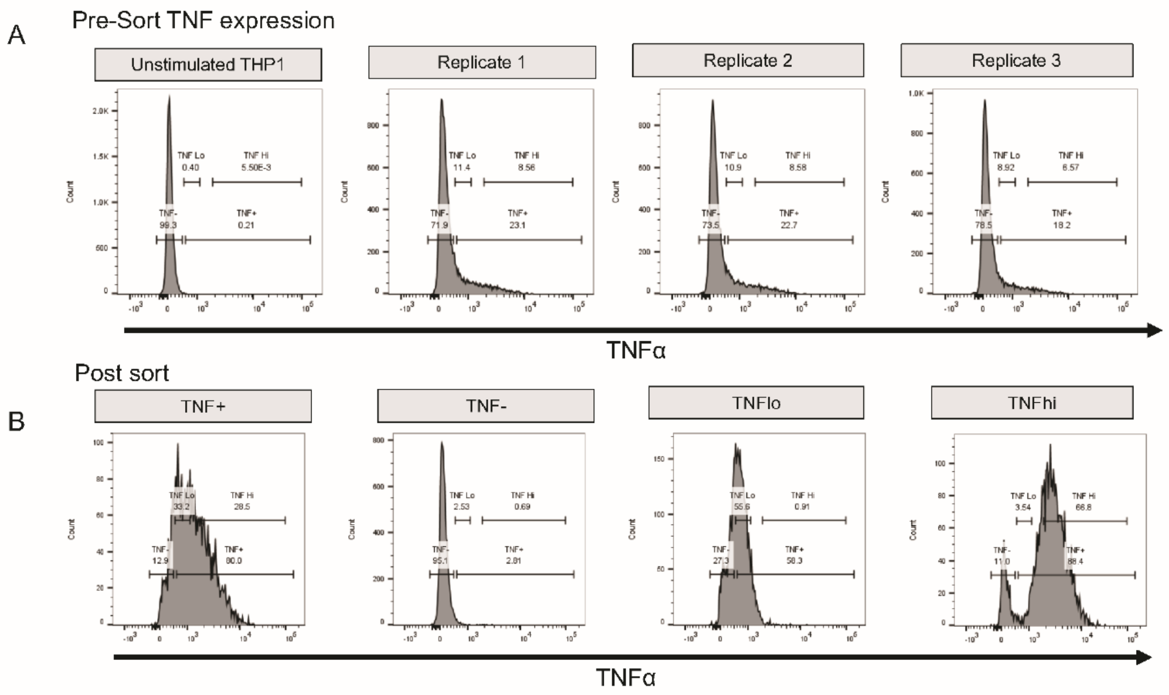

Supplemental Figure 2 – *TLR4* and *IRF8* sgRNAs positively correlate with LPS-induced TNFα levels in sorted THP1 populations. Log Fold change of sgRNAs targeting *TLR4* (A) and *IRF8* B) TSS in TNFα sorted populations as compared to presort THP1 cells.

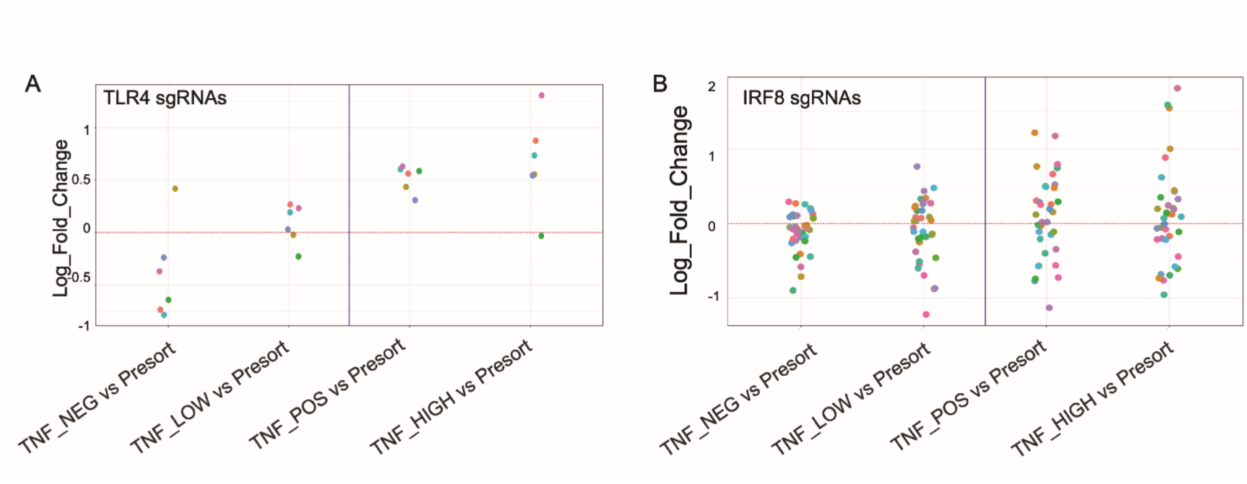

Supplement: Supplemental data [file jciinsight-9-168988-s059.pdf]
